# An explainable dual-branch transformer–ConvNeXt framework for robust lung histopathology classification

**DOI:** 10.3389/fonc.2026.1907746

**Published:** 2026-09-11

**Authors:** G. Sunitha, J. Vellingiri

**Affiliations:** School of Computer Science Engineering and Information Systems, Vellore Institute of Technology, Vellore, India

**Keywords:** LC25000, vision transformer, ConvNeXt, dual-branch fusion, metaheuristic feature selection, Monte-Carlo dropout, model calibration, explainable AI

## Abstract

**Introduction:**

Accurate histopathological discrimination of lung adenocarcinoma (ACA) and squamous cell carcinoma (SCC) from benign tissue is a clinically demanding task in which the two malignant subtypes are frequently confused even by trained pathologists.

**Methods:**

We present a dual-branch deep learning framework that couples a hierarchical shifted-window vision transformer (SwinV2-CR-Tiny) with a fully-convolutional masked-autoencoder-pretrained convolutional network (ConvNeXtV2-Tiny) to capture complementary global-contextual and local-textural representations of haematoxylin-and-eosin (H&E) stained tissue. The two branches are integrated through a learnable cross-feature gating module, refined by feature-space gated-modulation heads, and compressed by an Electric-Eel-Foraging-Optimizer (EEFO)-driven feature-selection stage. A Crested-Porcupine-Optimizer (CPO) calibrates class weighting and a Fennec-Fox-Optimizer (FFO) tunes per-class decision thresholds, while training combines Macenko stain normalization, CLAHE, MixUp/CutMix augmentation, a focal loss with label smoothing, Sharpness-Aware Minimization (SAM) and Stochastic Weight Averaging (SWA). Predictive uncertainty is quantified by Monte-Carlo (MC) dropout and model decisions are explained with Grad-CAM++ and Integrated Gradients. The model was evaluated on a stratified 70/15/15 partition of the LC25000 lung cohort (15,000 images; 5,000 per class).

**Results:**

The proposed model attains a test accuracy of 94.04%, macro-F1 of 0.9404, macro one-vs-rest AUC of 0.9886 and Cohen's κ of 0.9107, with near-perfect benign separation (sensitivity 0.999) and the residual error concentrated in the clinically expected ACA↔SCC confusion. Contrary to our prior expectation, MC-dropout predictive uncertainty did not separate misclassified from correctly classified cases on this cohort (mean across-pass standard deviation 0.0261 for errors versus 0.0269 for correct predictions). We additionally report a negative calibration finding: the model is systematically under-confident and naive temperature scaling increased the expected calibration error.

**Discussion:**

The negative uncertainty result is attributed to the same confidence-suppressing training objective and is reported rather than omitted. We further discuss the data-provenance characteristics of LC25000 that constrain the interpretation of absolute accuracy. The framework offers an interpret-able, transparently-evaluated decision-support pipeline whose error profile aligns with established lung-cancer histomorphology.

## 1 Introduction

Lung cancer remains the leading cause of cancer-related mortality worldwide, and the histopathological examination of resected or biopsied tissue stained with hematoxylin and eosin (H&E) is the diagnostic reference standard for subtyping non-small-cell lung carcinoma. The two dominant malignant subtypes, adenocarcinoma (ACA) and squamous cell carcinoma (SCC), require different therapeutic strategies, yet their morphological boundaries overlap considerably: poorly differentiated tumors may lose the glandular architecture characteristic of ACA or the keratinization and intercellular bridges characteristic of SCC, producing genuine inter-observer disagreement. Benign pulmonary parenchyma, by contrast, exhibits preserved alveolar architecture and is comparatively straightforward to distinguish. An automated decision-support system that reproduces this clinically meaningful error structure—reliably separating malignant from benign tissue while flagging the harder ACA↔SCC distinction with calibrated uncertainty would have direct value in high-throughput or resource-constrained pathology workflows.

Deep convolutional neural networks and, more recently, vision transformers have achieved expert-level performance on a range of computational-pathology tasks. Convolutional architectures excel at modeling local texture—nuclear chromatin patterns, cytoplasmic granularity and stromal organization—owing to their strong locality and translation-equivariance inductive biases. Transformer architectures, through self-attention, model long-range spatial dependencies and global tissue context that convolutions capture only at deep layers. These inductive biases are complementary rather than redundant, which motivates hybrid designs that exploit both. At the same time, three practical obstacles continue to limit the clinical translation of such models: (i) staining and scanner variability across laboratories degrades generalization; (ii) point predictions without calibrated confidence are unsafe for autonomous clinical use; and (iii) opaque “black-box” decisions are poorly suited to a regulated diagnostic setting that demands human-auditable evidence.

This work addresses all three obstacles within a single, reproducible pipeline evaluated on the publicly available LC25000 lung cohort. We design a dual-branch backbone that fuses a SwinV2-CR-Tiny transformer with a ConvNeXtV2-Tiny convolutional network, the latter pretrained with a fully-convolutional masked autoencoder (FCMAE). The two 768-dimensional embedding streams are combined through a learnable cross-feature gating module and refined by lightweight gated-modulation heads operating in the pooled feature space. To control representation redundancy and class imbalance and to optimize the operating point of the classifier, we incorporate three population-based metaheuristic optimizers at distinct, well-defined stages: the Electric-Eel-Foraging Optimizer (EEFO) selects a discriminative feature subset, the Crested-Porcupine Optimizer (CPO) tunes class weights, and the Fennec-Fox Optimizer (FFO) calibrates per-class decision thresholds. Robust optimization is achieved through stain normalization, focal loss with label smoothing, Sharpness-Aware Minimization (SAM) and Stochastic Weight Averaging (SWA). Finally, Monte-Carlo (MC) dropout supplies per-case epistemic uncertainty and a Grad-CAM++/Integrated-Gradients explainability layer renders the model auditable.

A distinguishing feature of this study is its commitment to honest reporting. We present not only the favorable discrimination metrics but also a negative calibration result—the trained network is systematically under-confident and naive temperature scaling, fitted to negative log-likelihood, increased rather than decreased the expected calibration error. We further discuss the data-provenance properties of LC25000, which are known to complicate the interpretation of absolute accuracy figures reported on this benchmark. We believe such transparency is essential for any model intended to inform clinical decisions.

Methodologically, the design choices in this work are deliberately conservative and composable rather than novel in isolation: each ingredient—dual-stream backbones, gated fusion, metaheuristic search, sharpness-aware and weight-averaged optimization, MC-dropout uncertainty and gradient-based attribution—is individually established, and our contribution lies in their disciplined integration into a single reproducible pipeline together with a transparent accounting of what does and does not work. We regard this transparency as a feature of the contribution rather than a hedge: a decision-support model whose limitations are precisely characterized is more useful to a clinical audience than one whose headline accuracy is unaccompanied by calibration, uncertainty or provenance analysis. The sections that follow therefore foreground not only the strong discrimination achieved but also the calibration failure, the incomplete internal benchmark, and the dataset-provenance caveat, so that the reader can weigh the evidence accurately.

### 1.1 Contributions

The principal contributions of this work are summarized as follows:

1. A complementary dual-branch backbone. We combine a hierarchical shifted-window transformer (SwinV2-CR-Tiny) and an FCMAE-pretrained convolutional network (ConvNeXtV2-Tiny), each yielding a 768-dimensional embedding, and integrate them through a learnable cross-feature gating mechanism that adaptively balances each branch’s self-representation against information attended from the other branch.
2. A metaheuristic-assisted decision pipeline. Three population-based optimizers are deployed at clearly delimited stages—EEFO for feature-subset selection, CPO for class-weight calibration and FFO for per-class threshold tuning—each formulated with an explicit fitness function and search space.
3. A robust, stain-aware training regime. Macenko normalization and CLAHE standardize color and contrast; MixUp and CutMix regularize the decision surface; and a focal loss with label smoothing, combined with SAM and SWA, drives the network toward flat, well-generalizing minima.
4. Uncertainty quantification with an honest calibration analysis. MC-dropout supplies per-case predictive uncertainty; we report two negative findings rather than omitting them—naive temperature scaling worsened calibration, and the MC-dropout uncertainty did not separate correct from incorrect predictions on this cohort—and we analyze both, tracing them to a common confidence-suppressing training objective.
5. Multi-method explainability. Grad-CAM++ and Integrated Gradients localize the morphological evidence underlying each prediction, supporting human-auditable, clinically interpretable outputs.
6. A transparent evaluation. We report a comprehensive metric suite (accuracy, macro-F1, AUC, Cohen’s κ, per-class sensitivity/specificity, calibration error and predictive uncertainty), benchmark against published LC25000 lung studies, and explicitly document dataset-provenance and protocol limitations.

The remainder of the work is organized as follows. Section 2 reviews related work in computational lung histopathology, hybrid CNN–transformer architectures, metaheuristic optimization and trustworthy medical AI. Section 3 describes the dataset and materials. Section 4 details the methodology, including all governing equations and algorithmic procedures. Section 5 specifies the model parameters, implementation and evaluation metrics. Section 6 reports and discusses the results. Sections 7–10 cover clinical applicability, comparative analysis, limitations and future work, and Section 11 concludes.

## 2 Related work

### 2.1 Deep learning for lung and colon histopathology

Deep learning has reshaped computational pathology over the past decade. Comprehensive surveys (2, 3) document the migration from handcrafted texture descriptors to end-to-end convolutional representations across detection, segmentation and classification tasks. In the specific context of lung tissue, Coudray et al. (4) demonstrated that a deep network trained on whole-slide images could not only classify ACA versus SCC versus normal lung but also predict driver-gene mutations directly from morphology, establishing histopathology images as a rich substrate for deep representation learning. Esteva et al. (5), although addressing dermatology, provided an influential template for dermatologist-level diagnostic performance from convolutional networks and motivated subsequent clinical-grade pathology systems.

The LC25000 dataset introduced by Borkowski et al. (1) has become a widely used public benchmark for lung and colon tissue classification, comprising 25,000 color images across five classes (three lung, two colon), each derived through augmentation from a smaller set of original tiles. Numerous studies have reported very high accuracies—frequently in the 96–100% range—on the lung subset using transfer-learned convolutional backbones and ensemble strategies (6, 7). As we discuss in Section 9, the augmentation-based construction of LC25000 has important implications for how such figures should be interpreted, and several of these reports do not control for the provenance of augmented tiles when partitioning data.

### 2.2 Hybrid CNN–transformer architectures

The vision transformer (ViT) (8) reframed image recognition as sequence modeling over patch embeddings, while the Swin Transformer family (9) reintroduced hierarchy and locality through shifted-window self-attention, achieving favorable accuracy–efficiency trade-offs at high resolution. In parallel, the ConvNeXt line modernized pure convolutional networks, and ConvNeXtV2 (10) coupled the architecture with fully-convolutional masked-autoencoder pretraining to yield strong transferable features. Because convolutional and attention-based models encode different inductive biases—locality and translation-equivariance versus global context—hybrid and dual-stream designs that combine them have repeatedly outperformed either family alone on medical imaging tasks. Our framework follows this principle but, rather than interleaving convolution and attention within a single tower, maintains two independent pretrained towers and fuses their pooled embeddings through a learnable gating module, preserving the distinct representational character of each branch.

### 2.3 Stain normalization and augmentation in histopathology

Inter-laboratory variability in H&E staining and digitization is a primary cause of distribution shift in computational pathology. The stain-separation method of Macenko et al. (11) decomposes optical-density images onto a reference hematoxylin–eosin basis and remains a standard normalization baseline. Contrast-limited adaptive histogram equalization (CLAHE) (12) complements color normalization by enhancing local nuclear contrast. On the augmentation side, MixUp (13) and CutMix (14) regularize the decision boundary by training on convex and spatially-spliced combinations of samples and labels, and label smoothing (15) mitigates over-confidence. We integrate these established techniques into a unified preprocessing and augmentation stage.

### 2.4 Robust optimization: SAM, SWA and focal learning

Generalization in over-parameterized networks is closely tied to the flatness of the converged minimum. Sharpness-Aware Minimization (SAM) (16) explicitly minimizes a perturbed worst-case loss within a neighborhood of the weights, while Stochastic Weight Averaging (SWA) (17) averages weights along the training trajectory to locate wider optima. Focal loss (18), originally proposed for dense object detection, down-weights easy examples and focuses learning on hard cases; combined with label smoothing it both addresses class difficulty and tempers confidence. Our training regime composes all of these.

### 2.5 Metaheuristic optimization for model and feature design

Population-based metaheuristics provide gradient-free search over discrete or non-differentiable design spaces such as feature subsets and decision thresholds. A recent wave of bio-inspired algorithms has been applied to such problems, including the Crested Porcupine Optimizer (CPO) (19), the Electric-Eel-Foraging Optimizer (EEFO) (20) and the Fennec-Fox Optimization algorithm (FFO) (21). In this work we use EEFO to search a binary feature-selection mask, CPO to tune class weights and FFO to optimize per-class decision thresholds. We restrict our claims to these three optimizers, each of which is concretely instantiated with an explicit fitness function (Section 4); we do not claim contributions from optimizers that are not actually invoked in the reported configuration.

### 2.6 Trustworthy medical AI: uncertainty, calibration and explainability

Clinical deployment requires more than accurate point predictions. Monte-Carlo dropout (22) approximates Bayesian inference by performing multiple stochastic forward passes, yielding a tractable estimate of predictive uncertainty that supports selective prediction and human referral. Calibration—the agreement between predicted confidence and empirical accuracy—is commonly assessed by the expected calibration error (ECE) and improved *post hoc* by temperature scaling (23); however, as our results demonstrate, temperature scaling is not universally beneficial and can worsen calibration when a model is under- rather than over-confident. For explainability, gradient-based attribution methods such as Grad-CAM++ (24) and Integrated Gradients (25), together with Shapley-value approaches (26), localize the image evidence underlying a prediction. Manifold-learning projections such as t-SNE (27) and UMAP (28) provide a complementary, global view of the learned feature geometry. Our framework operationalizes all of these trustworthiness components.

### 2.7 Positioning of the present work

Against this backdrop, three gaps motivate our design. First, the great majority of LC25000 lung studies optimize raw accuracy with a single transfer-learned backbone and report little or nothing on calibration, predictive uncertainty or provenance-aware evaluation, leaving their high accuracies difficult to act on clinically. Second, where hybrid CNN–transformer models are used, the two representational families are usually entangled within one tower, obscuring the individual contribution of each; we instead keep two independent pretrained towers and fuse their pooled embeddings, which makes the dual-branch benefit directly ablatable. Third, the trustworthiness components—uncertainty, calibration and explainability—are typically reported, if at all, in isolation rather than as an integrated and mutually-consistent stack. The present work addresses the first and third gaps by composing all trustworthiness components into one pipeline and reporting them jointly (including a negative calibration result), and sets up the second as an explicit, if currently unfinished, ablation. We do not claim novelty for the individual components; we claim a transparent, reproducible integration and an honest evaluation that is itself in short supply on this benchmark.

## 3 Materials and dataset

### 3.1 The LC25000 lung cohort

All experiments use the lung subset of the publicly available LC25000 histopathological image dataset (1). The lung subset contains 15,000 color images of H&E-stained tissue, evenly distributed across three diagnostic classes—lung adenocarcinoma (ACA), benign lung tissue and lung squamous cell carcinoma (SCC)—with exactly 5,000 images per class. Each image is an RGB tile of 768×768 pixels, which we resize to 224×224 to match the native input resolution of both backbones. The dataset is fully de-identified and HIPAA-compliant as released by its authors. the visual distinctions—glandular and papillary architecture in ACA, preserved thin-walled alveolar spaces with capillary congestion in benign tissue, and densely packed sheets of pleomorphic keratinizing cells in SCC—are evident even to a non-specialist, while the ACA–SCC boundary is visibly the most subtle.

### 3.2 Data partitioning

The cohort is divided into mutually exclusive training, validation and test partitions using a two-step stratified shuffle split that preserves the exact class proportions in every partition. A first split allocates 70% of images to training; the remaining 30% is split equally into validation and test sets, yielding a final 70/15/15 partition of 10,500/2,250/2,250 images, i.e. 3,500 training and 750 validation and 750 test images per class. A fixed random seed (*seed = 42*) governs all stochastic operations—partitioning, weight initialization, augmentation sampling and optimizer search—so that the entire pipeline is reproducible. The class-balanced partition is summarized in **Table 1**.

**Table 1:** LC25000 lung dataset composition and stratified 70/15/15 partition.

Class	Label	Train	Validation	Test	Total
Adenocarcinoma (ACA)	0	3,500	750	750	5,000
Benign	1	3,500	750	750	5,000
Squamous cell carcinoma (SCC)	2	3,500	750	750	5,000
Total	**—**	**10,500**	**2,250**	**2,250**	**15,000**

The bold values indicate the overall totals for the train, validation, test, and complete dataset.

## 4 Methodology

**Figure 1:**
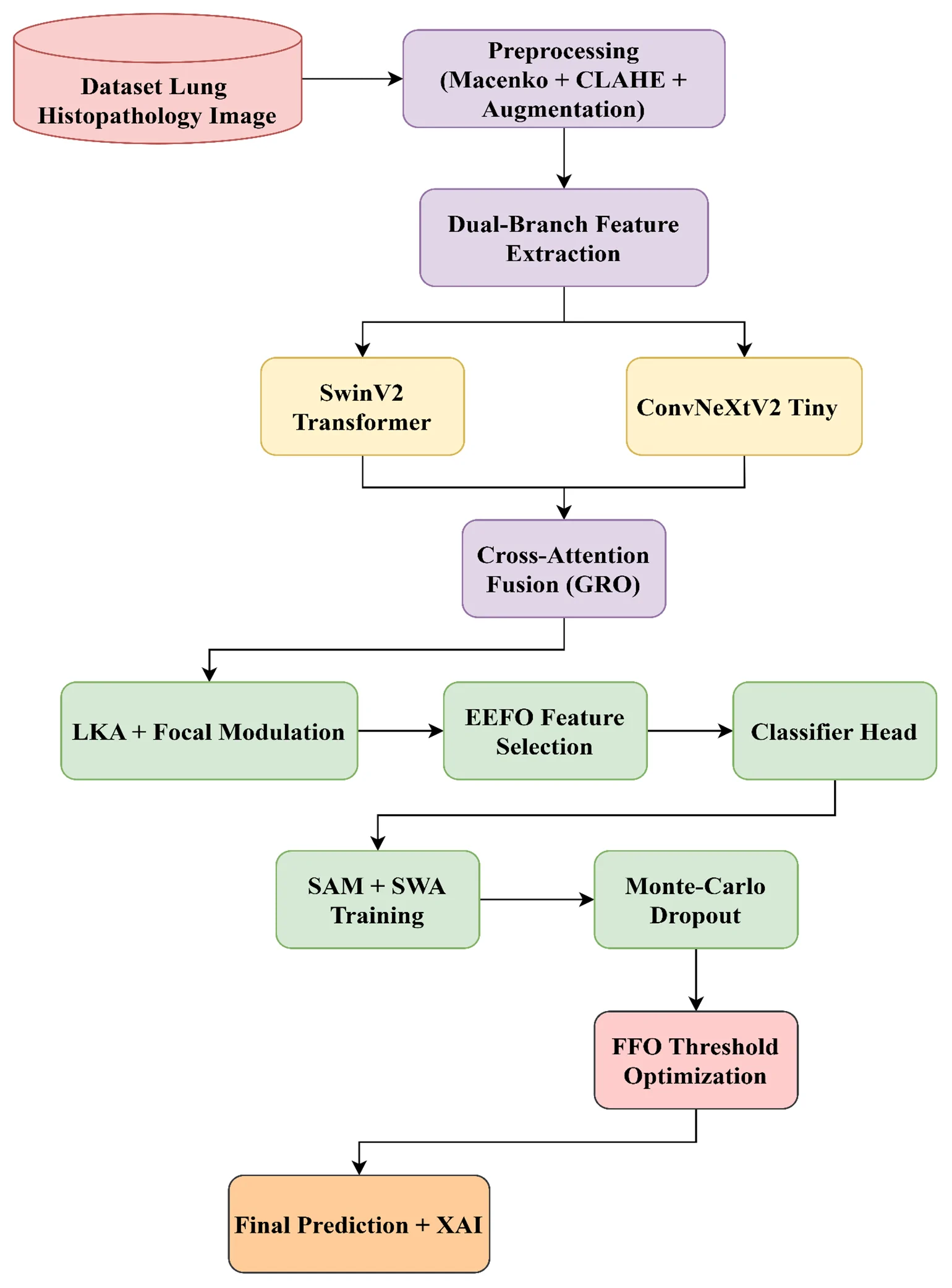
Overall workflow of the proposed architecture, illustrating the sequential stages of image preprocessing, feature extraction, feature selection, classification, uncertainty quantification, and explainability analysis.

**Figure 2:**
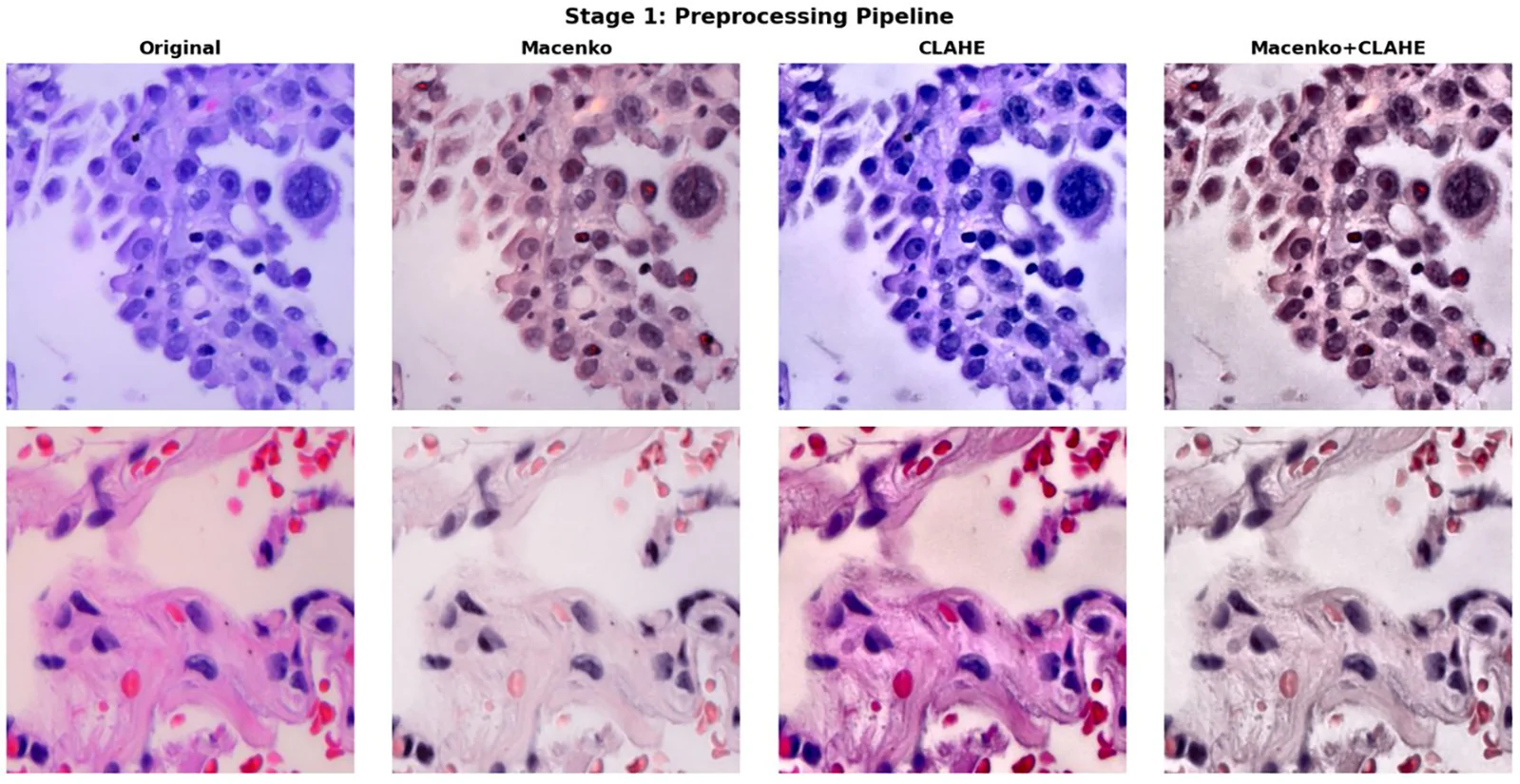
Stage-1 preprocessing. Columns show the original tile, Macenko-normalized output, CLAHE-enhanced output and the combined Macenko+CLAHE result for an ACA (top) and a benign (bottom) example. Normalization harmonizes the stain color distribution while CLAHE sharpens nuclear detail.

**Figure 3:**
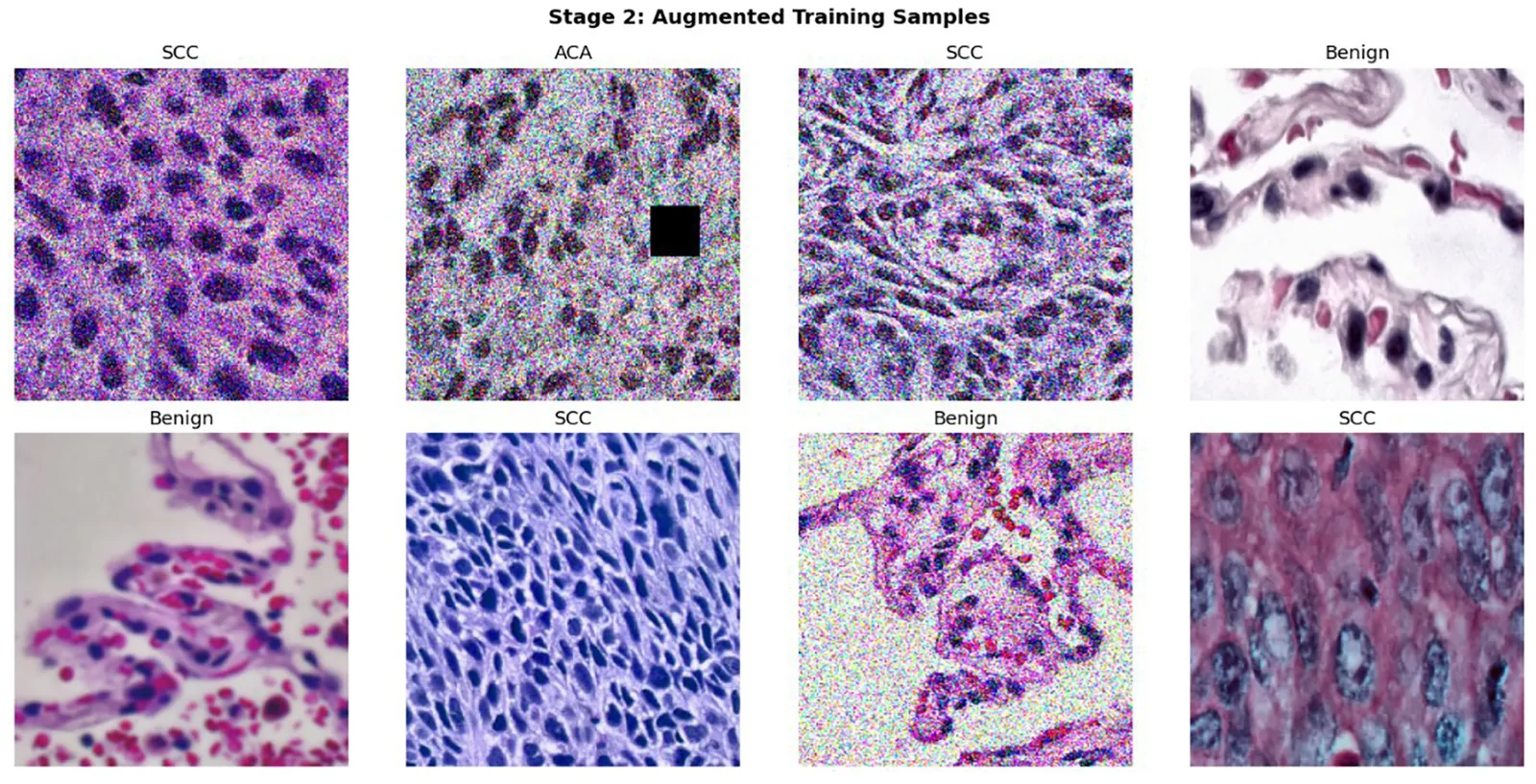
Stage-2 augmented training samples after the full geometric/photometric pipeline (and, where sampled, MixUp/CutMix). Strong perturbations enlarge the effective training distribution while preserving class-discriminative morphology.

The proposed pipeline comprises seven functional stages: (i) stain normalization and contrast enhancement; (ii) augmentation and class-weight calibration; (iii) dual-branch feature extraction; (iv) cross-feature gated fusion and feature-space refinement; (v) metaheuristic feature selection; (vi) robust training with focal learning, SAM and SWA; and (vii) *post-hoc* uncertainty quantification, calibration analysis, threshold optimization and explainability. **Figure 1** as shown in above architecture, **Figures 2**, **3** illustrate the preprocessing and augmentation stages; the remaining stages are formalized below. The principal notation used in this study is summarized in **Table N1**. Throughout, an input image is denoted x ∈ ℝ^(3×H×W), the ground-truth label y ∈ {0,1,2}, and the number of classes C = 3.

**Table N1:** Principal notation used throughout the methodology.

Symbol	Meaning	Symbol	Meaning
x, y	input image, ground-truth label	C	number of classes (= 3)
d	embedding dimension (= 768)	f_1_, f_2_	transformer/convolutional features
c_1_→_2_	cross-attended response	α, β	learnable fusion gates
z, zã	fused/refined representation	m	binary EEFO feature mask
w_c	CPO class weights	τ_c	FFO per-class thresholds
p, p̄	predictive/MC-averaged prob.	u	predictive uncertainty
T	temperature/MC passes	ρ	SAM radius/keep-ratio
γ, ϵ	focal focus/label smoothing	η	learning rate

End-to-end, the model defines a single differentiable map from a tile to a class distribution, with two non-differentiable search stages bolted on at clearly delimited points. A pre-processed tile x ∈ ℝ^(3×224×224) is passed in parallel to the two pretrained towers of Stage 3, each of which returns a global-average-pooled embedding of dimension d = 768; because the towers already agree in width, the inter-branch projection is an identity map and no information is lost at the interface. A dropout layer at rate 0.3 follows each embedding and is deliberately left active at inference to realize the Monte-Carlo-dropout estimator of Stage 7. The two embeddings enter the cross-feature gating module of Stage 4, where an eight-head query–key interaction lets each branch attend to the other and two learnable scalars α, β decide how much attended information to admit; the gated branches are concatenated, linearly projected back to 768 dimensions and layer-normalized to give the fused vector. This vector is refined by two parallel channel/feature-space heads—a gated-refinement head and a focal-modulation head—whose outputs are combined by two further learnable scalars, yielding the refined 768-dimensional representation. The Stage-5 EEFO mask, a fixed binary vector retaining 421 of the 768 channels, is then applied multiplicatively, and the surviving channels are mapped to three logits by the two-layer classifier Linear(768→512)→GELU→Dropout→Linear(512→3). All learnable parameters of the fusion, refinement and classifier blocks, together with both backbones, are trained jointly under the focal-plus-label-smoothing objective of Stage 6 using Sharpness-Aware Minimization over AdamW and a cosine-to-SWA schedule. The two threshold/weight searches—CPO for class weights (Stage 2) and FFO for decision thresholds (Stage 7)—and the EEFO feature search (Stage 5) operate on frozen, gradient-free objectives and therefore sit outside the back-propagation graph, which keeps the end-to-end training path clean while still allowing non-differentiable design choices to be optimized.

Design rationale. Three choices distinguish this pipeline from the more common single-tower hybrids and deserve explicit justification. First, the two representational families are kept in separate, independently pretrained towers rather than interleaved within one network: this preserves the distinct inductive biases of each—locality and translation-equivariance in the convolutional branch, global self-attention context in the transformer branch—and, equally important, makes the marginal value of the dual-branch design directly ablatable against either branch in isolation. Second, the fusion and refinement operate on pooled feature vectors, not on spatial maps; we therefore describe the refinement heads as channel/feature-space gated-modulation blocks and avoid the term spatial large-kernel attention, which would overstate their relationship to convolutional attention operators that act over a spatial grid. Third, the EEFO mask is selected on the features of a one-epoch warm-up model and then applied to a freshly re-initialized model that is trained from scratch (**Algorithm 2**); a consequence we state plainly is that the mask acts as a fixed structured bottleneck whose 421 retained channels are not guaranteed to be the semantically most informative ones of the final model. The decisive test of whether this bottleneck helps, hurts or is neutral is the without-EEFO ablation specified in Section 7.3, and we report it as an open experiment rather than asserting a benefit. These three positions reflect the work’s broader stance: every component is individually established, and the contribution is their disciplined, faithfully-reported integration.

Algorithm 1. Two-phase training of the proposed model

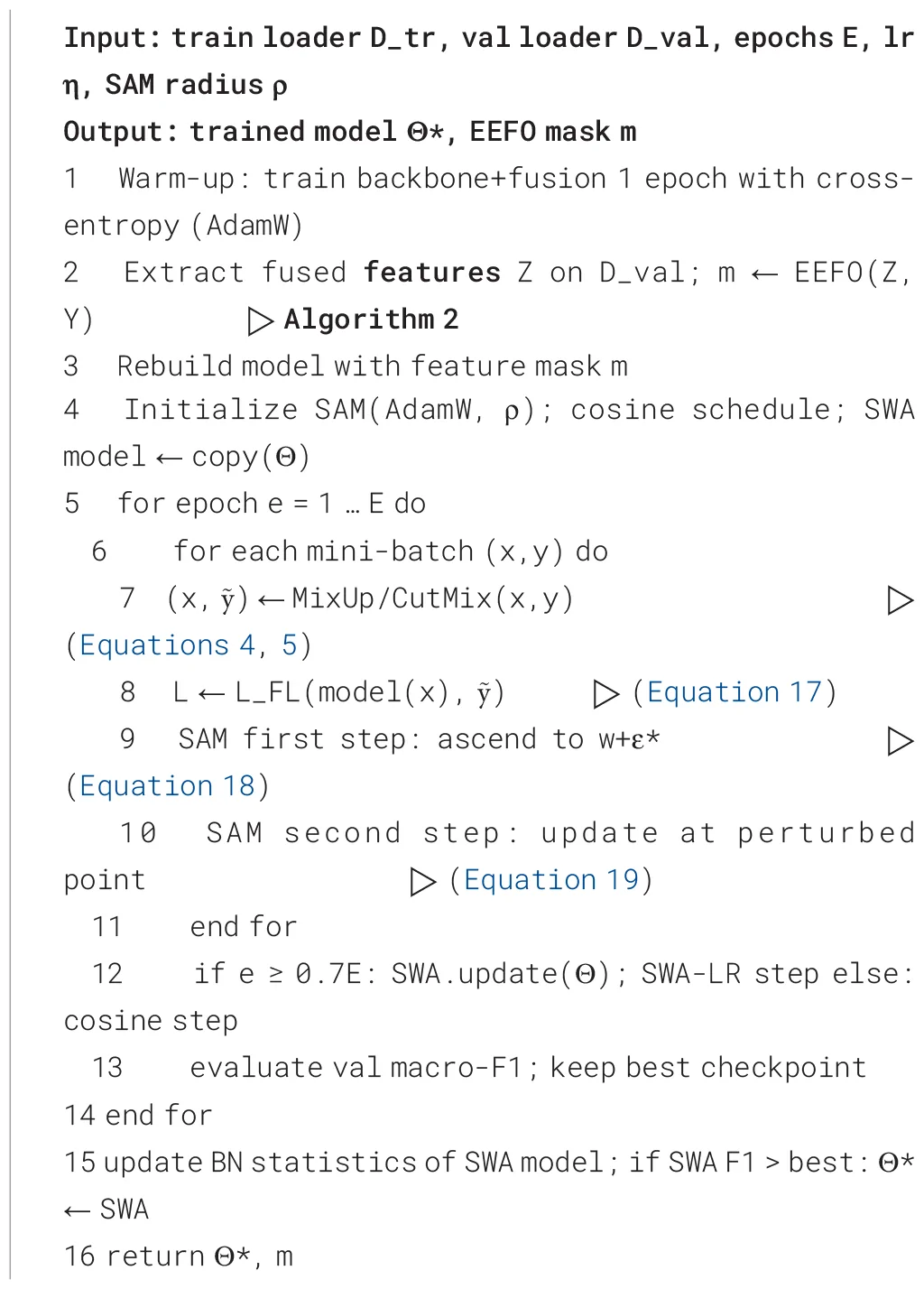

### 4.1 Stage 1 — Macenko stain normalization and CLAHE

To suppress inter-slide color variation, each tile is mapped onto a fixed reference H&E stain basis using the Macenko transform (11). Pixel intensities I are first converted to optical density (OD) is defined as Equation 1:

(1) $$OD=-\ln\left(I+1\right)/I_{0})$$

where *I*_0_ is the transmitted-light intensity (set to 240). Optical-density vectors below a threshold β are discarded as background, and the two principal stain directions are recovered as the eigenvectors of the OD covariance matrix corresponding to its two largest eigenvalues. Projecting the OD data onto this plane and taking robust angular percentiles (α and 100−α) yields the estimated stain matrix $\hat{H}E\in \mathbb{R}\left(3\times 2\right)$. The stain concentrations *C* are obtained by solving the non-negative least-squares system is defined as Equation 2:

(2) $$C=\arg\min\_\{C\geq 0\}{\left\|\hat{H}E\cdot C-OD^{\text{T}}\right\|}_{2}^{2}$$

and rescaled to a reference maximum concentration vector *maxC*_ref before reconstruction onto the reference basis $\hat{H}E\text{\_ref}$ as defined in Equation 3:

(3) $$I\text{\_norm}=I_{0}\cdot \exp\left(-\hat{H}E\text{\_ref}\cdot \left(C⊙\left(maxC\text{\_ref}/maxC\right)\right)\right)$$

After normalization, contrast-limited adaptive histogram equalization (CLAHE) (12) is applied to the luminance (L) channel of the CIE-Lab representation with a clip limit of 2.0 and an 8×8 tile grid, enhancing local nuclear contrast while bounding noise amplification. To balance fidelity and runtime, Macenko normalization is applied stochastically with probability 0.3 during training, whereas CLAHE is applied to every tile. **Figure 2** visualizes the effect of each operation.

### 4.2 Stage 2 — augmentation and CPO class-weight calibration

Geometric and photometric augmentation is performed with horizontal/vertical flips, 90° rotations, random resized cropping (scale 0.8–1.0), color jitter, hue–saturation–value perturbation, Gaussian blur and noise, and coarse dropout, followed by channel-wise normalization to ImageNet statistics. The full augmentation specification is given in **Table 2**. We deliberately use geometric and photometric augmentation in place of generative (e.g. diffusion-based) synthesis, which exceeds the compute budget and offers limited benefit on an already class-balanced cohort. Representative examples of the resulting Stage-2 augmented training samples are shown in **Figure 3**.

**Table 2:** Augmentation operations applied to the training stream (validation and test streams use only resize and normalization).

Operation	Parameters	Probability
Random resized crop	scale (0.8, 1.0), output 224×224	1.00
Horizontal flip	—	0.50
Vertical flip	—	0.50
Random 90° rotation	{0°,90°,180°,270°}	0.50
Color jitter	brightness/contrast/saturation 0.2, hue 0.05	0.50
Hue–saturation–value	± 10/± 15/± 10	0.50
Gaussian blur	kernel 3–5	0.20
Gaussian noise	default variance	0.20
Coarse dropout	rectangular occlusions	0.25
MixUp/CutMix	α_mix = 0.2, α_cut = 1.0	0.50/0.40

Sample-mixing augmentation is applied at the batch level. With probability 0.5 a MixUp (13) combination is formed according to Equation 4:

(4) $$\tilde{x}=\lambda \;x\text{\_i}+\left(1-\lambda \right)x\text{\_j},\;\tilde{y}=\lambda \;y\text{\_i}+\left(1-\lambda \right)y\text{\_j},\;\lambda \sim \text{Beta}\left(\text{α\_mix, α\_mix}\right)$$

and otherwise (with probability 0.4) a CutMix (14) combination splices a rectangular region B from a second image according to Equation 5.

(5) $$\tilde{x}=M⊙x\text{\_i}+\left(1-M\right)⊙x\text{\_j},\;\lambda =1-\left|\text{B}\right|/\left(\text{H W}\right)$$

where *M* is a binary box mask and the mixing coefficient λ weights the two label components. The mixed soft label $\tilde{y}=\lambda \cdot \text{oneshot}\left(y\text{\_i}\right)+\left(1-\lambda \right)\cdot \text{oneshot}\left(y\text{\_j}\right)$ is used by the loss in Equation 17.

Although the cohort is balanced, we include a Crested-Porcupine-Optimizer (CPO) (19) stage that searches a class-weight vector *w* ∈ [0.5, 2.0]^C minimizing the squared deviation from the inverse-frequency weighting *b* according to Equation 6:

(6) $$F\text{\_CPO}\left(w\right)=\sum \text{\_c}{\left(\hat{w}\text{\_c}-\text{b\_c}\right)}^{2},\hat{w}=w/\text{mean}\left(w\right),\;\text{b\_c}=\text{N}/\left(\text{C}\cdot \text{N\_c}\right)$$

On the balanced LC25000 cohort the converged weights are *w* = (0.998, 1.001, 1.002), confirming that no class re-weighting is warranted; the stage thus functions as an automated sanity check and would re-weight automatically on an imbalanced cohort.

### 4.3 Stage 3 — dual-branch feature extraction

The backbone comprises two pretrained towers operating in parallel on the same input. The transformer branch φ_swin is a SwinV2-CR-Tiny network with hierarchical shifted-window self-attention, chosen because the cross-resolution (CR) variant operates natively at 224×224. The convolutional branch φ_cnx is a ConvNeXtV2-Tiny network pretrained with a fully-convolutional masked autoencoder (FCMAE) (10). Each tower emits a global-pooled embedding of dimension *d* = 768 as defined in Equation 7:

(7) $$f_{1}=\varphi \text{\_swin}\left(x\right)\in {\mathbb{R}}^{\text{d}},\;f_{2}=\varphi \text{\_cnx}\left(x\right)\in {\mathbb{R}}^{\text{d}}$$

Because both towers already emit 768-dimensional features, the dimensionality-matching projection reduces to an identity map; in the general case a linear projection *W*_p aligns the transformer embedding to the convolutional dimension. A dropout layer (rate 0.3) is applied to each branch output to enable MC-dropout uncertainty estimation (Section 4.7).

### 4.4 Stage 4 — cross-feature gated fusion and refinement

The two embeddings are fused by a learnable cross-feature gating module. For each branch, a query is matched against the other branch’s key across *h* = 8 heads. Writing the per-head query, key and value as *q*^(h), *k*^(h), *v*^(h) ∈ ℝ^(d/h), the cross-attended response from branch 2 to branch 1 is defined as Equations 8, 9:

(8) s^(h)=(q1^(h)⊙k2^(h))·1/√(d/h),a^(h)=softmax_h(s^(h))

(9) c1→c2=∑_h a^(h) v2^(h), c2→1=∑_h a˜^(h) v1^(h)

where the softmax is taken across heads. The fused per-branch representations interpolate between each branch’s own features and the information attended from the complementary branch, governed by learnable scalars α, β ∈ [0,1] as defined in Equations 10, 11:

(10) $$g_{1}=\alpha \;f_{1}+\left(1-\alpha \right)\;c_{1}\to_{2},\;g_{2}=\beta \;f_{2}+\left(1-\beta \right)\;c_{2}\to_{1}$$

(11) $$z=\text{LayerNorm}\left(W\text{\_p}\;[g_{1};g_{2}]\right)\in {\mathbb{R}}^{\text{d}}$$

We emphasize, for accuracy, that α and β are optimized end-to-end by back-propagation rather than by an external optimizer in the reported configuration. The fused vector *z* is then refined by two parallel feature-space heads. A gated-refinement head computes a sigmoid gate over the channel dimension, as defined in Equation 12:

(12) $$r=W_{2}\;(\text{GELU}\left(W_{1}\;z\right)⊙\sigma \left(W\text{\_g }z\right))+z$$

and a channel focal-modulation head aggregates *L* = 3 context projections plus a global-context term through input-dependent gates *γ* = softmax(*W*_γ *z*) as define in Equation 13:

(13) mod=∑_{1=0}^{L} γ_1·ctx_1(z), o=W_o (q(z)⊙mod)+z

The two refined responses are combined by learnable weights γ_1_, γ_2_ to give the refined representation $\tilde{z}=\gamma_{1}\;r+\gamma_{2}\;o$. These heads operate on the pooled feature vector rather than on spatial maps; we therefore describe them as channel/feature-space gated-modulation blocks to avoid overstating their relationship to spatial large-kernel-attention operators.

### 4.5 Stage 5 — EEFO feature-subset selection

To reduce redundancy in the 768-dimensional fused representation, a binary selection mask *m* ∈ {0,1}^d^ is applied multiplicatively, *zã*’ = *zã* ⊙ *m*. The mask is searched by the Electric-Eel-Foraging Optimizer (EEFO) (20) on a held-out feature matrix. Each candidate real-valued vector *v* ∈ [0,1]^d^ is binarized at 0.5, and its fitness is the validation error of an ℓ_2_-regularized logistic-regression probe trained on the masked features, with a soft penalty steering the cardinality toward a target keep-ratio ρ = 0.75 as defined in Equation 14:

(14) $$F\text{\_EEFO}\left(m\right)=1-\text{Acc\_val}\left(\text{LR}\left(Z\text{\_tr}⊙m\right)\right)+\zeta {\left({\left\|m\right\|}_{0}/\text{d}-\rho \right)}^{2}$$

with ζ = 0.05 and a minimum of 16 retained features. Features are standardized before the probe is fit. The EEFO position update alternates exploration and exploitation governed by a decaying electric-field term *E* = *E*_0_ (1 − *t*/*T*) as defined in Equation 15:

(15) $${\text{v\_i}}^{\prime }=v^{*}+E\cdot \left|v^{*}-v\text{\_i}\right|⊙\epsilon ,\;\epsilon ∼N\left(0,\text{I}\right)\;(\text{exploitation, }\left|\text{E}\right|<1)$$

**Figure 4:**
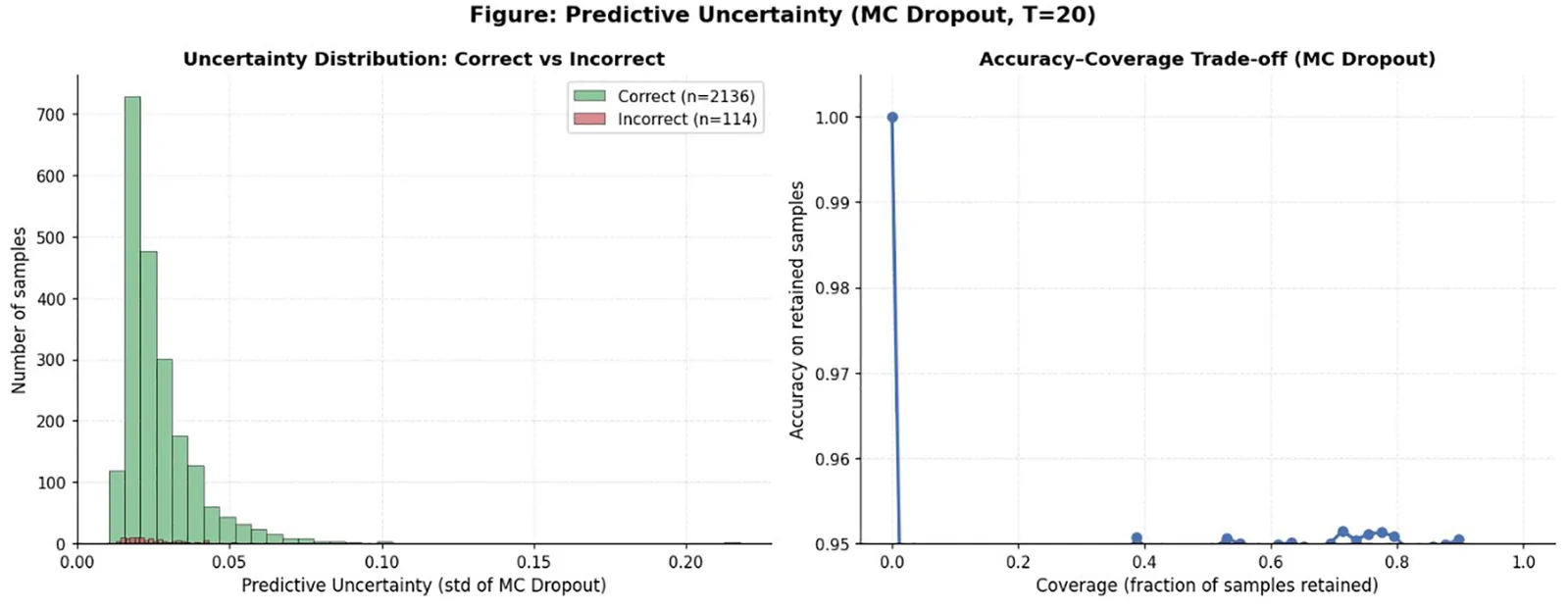
Monte-Carlo dropout uncertainty (T = 20). Left: distribution of predictive uncertainty for correct vs. incorrect predictions. Right: accuracy–coverage trade-off when low-confidence cases are deferred. Uncertainty cleanly separates errors and enables selective prediction.

On the LC25000 fused features, EEFO retains 421 of 768 dimensions (54.8%) at a converged probe fitness of 0.0395 (**Figure 4**, left). Algorithm 2 details the procedure.

### 4.6 Stage 6 — classifier, focal learning, SAM and SWA

The masked representation is mapped to class logits by a two-layer head with a GELU non-linearity and dropout as defined in Equation 16:

(16) $${\tilde{z}}^{\prime }=\left(\tilde{z}⊙m\right),\;\ell =W_{2}\cdot \text{Dropout}\left(\text{GELU}\left(W_{1}{\tilde{z}}^{\prime }\right)\right),p=\text{softmax}\left(\ell \right)$$

Training minimizes a focal loss (18) with label smoothing (15). For a (possibly soft) target distribution *t* with smoothing ϵ = 0.05 and focusing parameter γ = 2.0, as defined in Equation 17:

(17) L_FL=−∑_c w_c·t_c·(1−p_c)∧γ·logp_c

Where *w*_c are the CPO class weights from Equation 6. Optimization uses Sharpness-Aware Minimization (SAM) (16) wrapped around AdamW. SAM first ascends to the worst-case point within an ℓ_2_ ball of radius ρ = 0.05, as defined in Equation 18:

(18) $$\epsilon^{*}\left(w\right)=\rho \cdot \text{∇\_w}\;L\left(w\right)/{\left\|\text{∇\_w}\;L\left(w\right)\right\|}_{2}$$

and then updates the base weights using the gradient evaluated at the perturbed point, thereby penalizing sharp minima as defined in Equation 19:

(19) $$w\leftarrow w-\eta \cdot \text{∇\_w}\;L\left(w+\epsilon^{*}\left(w\right)\right)$$

A cosine-annealed learning-rate schedule is used until the SWA phase. Stochastic Weight Averaging (SWA) (17) activates after 70% of training and averages the weights visited under a constant SWA learning rate, as defined in Equation 20:

(20) w_SWA=(1/K)∑_{k=1}∧{K} w_k

followed by a batch-normalization statistics update over the training stream. The validation macro-F1 selects the best checkpoint, and the SWA model replaces it if superior. The full two-phase training procedure, including the warm-up pass that supplies features to EEFO, is given in **Algorithm 1**.

Algorithm 2. EEFO-based feature-subset selection.

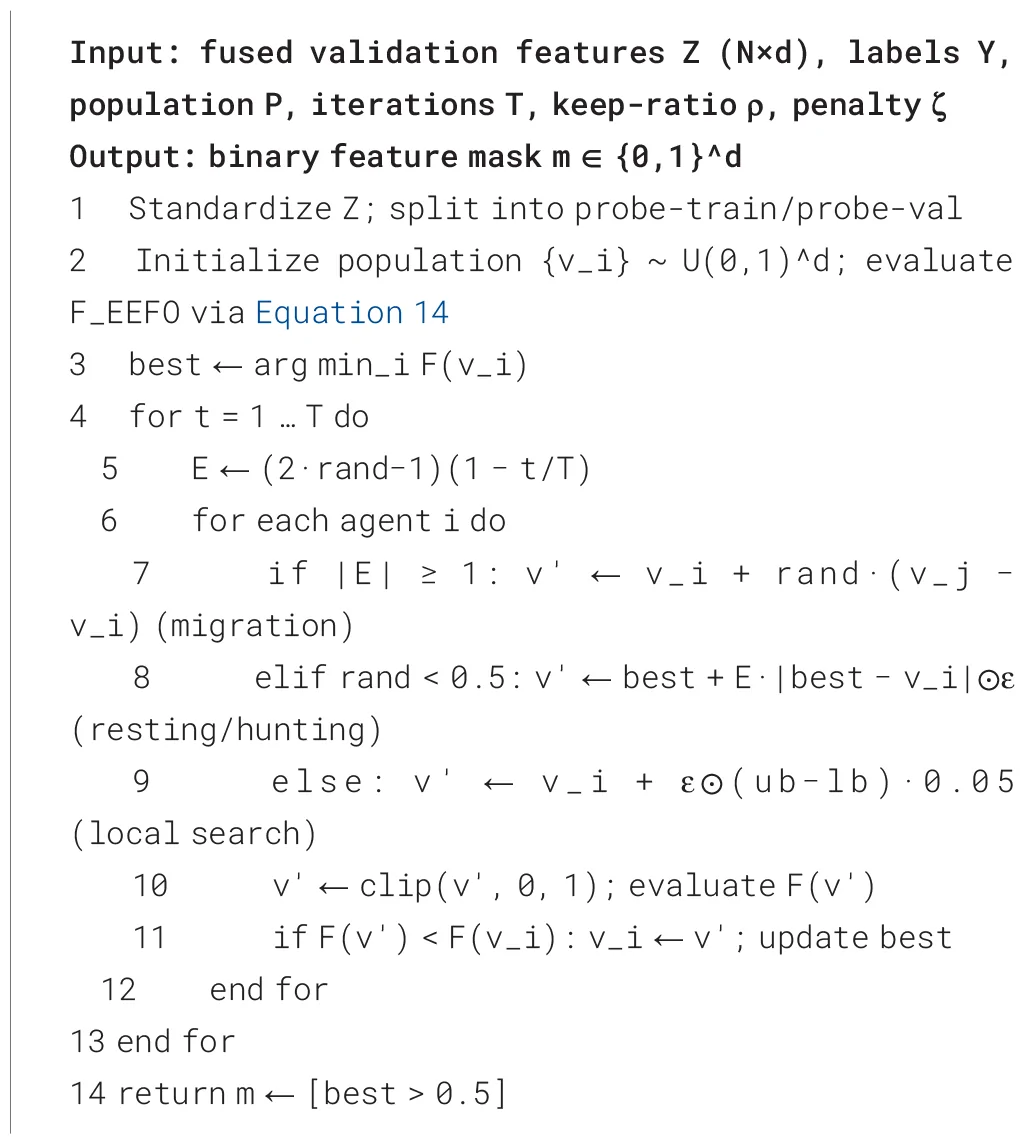

### 4.7 Stage 7 — uncertainty, calibration and FFO threshold tuning

Epistemic uncertainty is estimated by Monte-Carlo dropout (22). Keeping dropout active at inference, *T* = 20 stochastic forward passes are averaged, as defined in Equation 21:

(21) $$\bar{p}\left(x\right)=\left(1/\text{T}\right)\sum \_\{\text{t}=1\}\wedge \{\text{T}\}\text{ softmax}\left(\text{f\_}\{\text{Θ},\text{ drop\_t}\}\left(x\right)\right)$$

and the per-sample predictive uncertainty is the mean over classes of the across-pass standard deviation, as given in Equation 22:

(22) u(x)=(1/C)∑_c std_t(p_t, c(x))

Calibration is assessed with the expected calibration error (ECE) over *B* = 15 equal-width confidence bins, as defined in Equation 23:

(23) ECE=∑_{b=1}∧{B}(|B_b|/N)·|acc(B_b)−conf(B_b)|

and temperature scaling (23) is fitted on the validation logits by minimizing negative log-likelihood with respect to a single scalar T, after which probabilities are recomputed as p_T = softmax(ℓ/T). Finally, the Fennec-Fox Optimizer (FFO) (21) tunes a per-class additive decision threshold τ ∈ [−0.2, 0.2]^C to maximize validation macro-F1, as defined in Equation 24:

(24) $$\hat{y}=\arg\max\text{\_c}\left(\text{p\_\{T,c\}}-\tau \text{\_c}\right),\;F\text{\_FFO}\left(\tau \right)=1-\text{F}1\text{\_macro}\left(y,\hat{y}\right)$$

The complete inference-time post-processing is summarized in **Algorithm 3**.

Algorithm 3. Uncertainty quantification, calibration and FFO threshold optimisation.

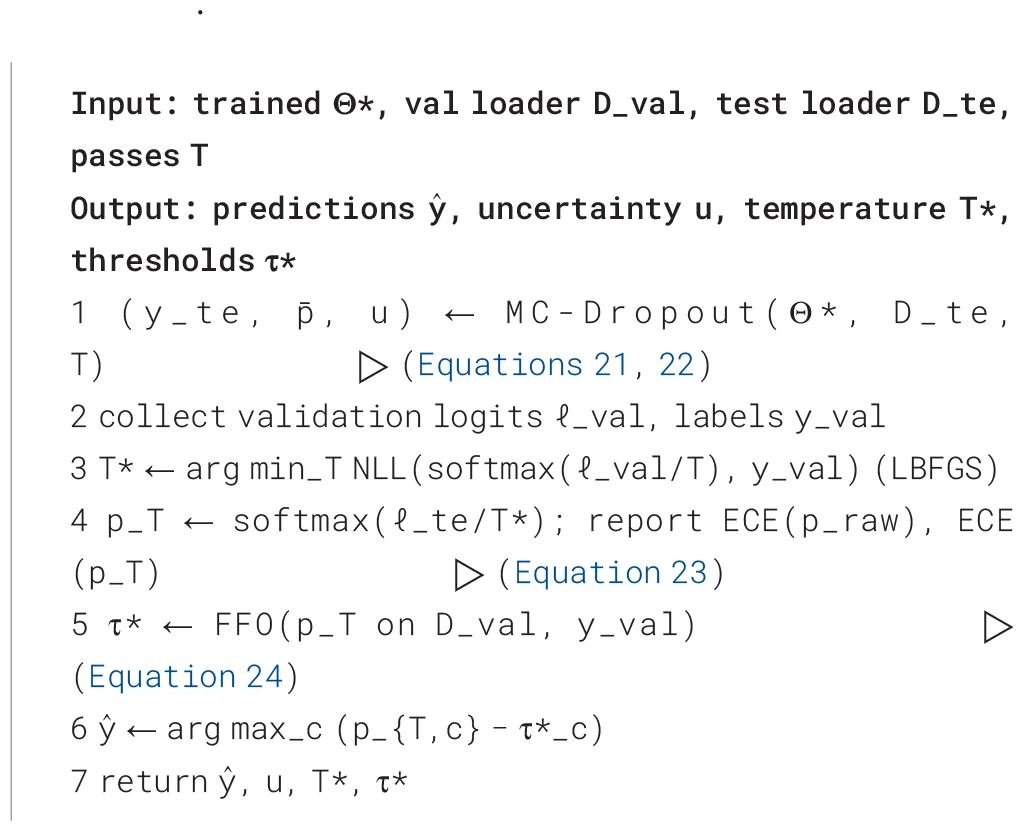

### 4.8 Stage 8 — explainability

Two complementary attribution methods render the model auditable. Grad-CAM++ (24) is applied to the final stage of the convolutional branch to produce class-discriminative spatial saliency maps, while Integrated Gradients (25) attributes the prediction to individual input pixels by integrating gradients along a path from a baseline. The resulting heat-maps (Section 6.7) localize the morphological evidence used by the model and allow a pathologist to verify that predictions rest on diagnostically relevant regions. Manifold projections (t-SNE (27), UMAP (28)) of the learned embeddings provide a complementary global view of class separability.

## 5 Model parameters, implementation and evaluation metrics

### 5.1 Architecture and complexity

The complete model contains 64.98 million trainable parameters. The two backbones contribute the majority of the parameter count, with the fusion module, refinement heads and classifier adding a comparatively small overhead. A theoretical floating-point operation (FLOP) count obtained with fvcore returned 9.14 GFLOPs; however, this figure is an under-estimate because several transformer and modulation operators (e.g. residual subtraction, padding, vector norm, softmax and GELU) are not enumerated by the profiler, as evidenced by the “unsupported operator” diagnostics emitted at counting time. The reported FLOP value should therefore be treated as a lower bound and re-measured with an operator-complete profiler before publication. **Table 3** lists the architectural configuration.

**Table 3:** Architecture configuration of the proposed dual-branch model.

Component	Specification	Output dim.
Transformer branch	SwinV2-CR-Tiny (swinv2_cr_tiny_ns_224), ImageNet-pretrained	768
Convolutional branch	ConvNeXtV2-Tiny (FCMAE, IN-22k→IN-1k)	768
Branch dropout	rate 0.3 (MC-dropout enabled)	—
Cross-feature gating	8 heads; learnable α, β; LayerNorm; linear projection	768
Refinement heads	gated-refinement + channel focal-modulation; learnable γ_1_,γ_2_	768
EEFO feature mask	binary mask, 421/768 retained	421
Classifier	Linear(768→512) → GELU → Dropout → Linear(512→3)	3
**Total parameters**	**64.98 M trainable**	**—**

### 5.2 Training hyperparameters

All hyperparameters are fixed *a priori* and reported in full in **Table 4** to support reproducibility. The proposed model is trained for 12 epochs; baselines and ablation variants use reduced budgets of 6 and 8 epochs respectively to control compute.

**Table 4:** Complete training and optimizer configuration.

Hyperparameter	Value	Hyperparameter	Value
Input resolution	224×224	Optimizer	SAM + AdamW
Batch size	32	Base learning rate	3×10^-4^
Epochs (proposed)	12	Weight decay	1×10^-4^
Epochs (baseline)	6	LR schedule	Cosine annealing
Epochs (ablation)	8	SAM radius ρ	0.05
Focal γ	2.0	SWA start fraction	0.70
Label smoothing ϵ	0.05	SWA learning rate	3×10^-5^
MixUp α	0.20	MC-dropout passes T	20
CutMix α	1.00	Calibration bins	15
Dropout rate	0.30	Random seed	42

### 5.3 Metaheuristic search configuration

**Table 5** lists the search configuration of the three metaheuristic optimizers that are actually invoked in the reported pipeline. We do not tabulate optimizers that are defined in the code base but not executed in this configuration, in the interest of accurate reporting.

**Table 5:** Configuration of the metaheuristic optimizers used in the reported pipeline.

Optimizer	Pipeline role	Search dim.	Bounds	Pop.	Iter.
CPO (19)	Class-weight calibration	3	[0.5, 2.0]	8	15
EEFO (20)	Feature-subset selection	768	[0, 1]	12	25
FFO (21)	Per-class threshold tuning	3	[−0.2, 0.2]	10	20

### 5.4 Hardware and software environment

Experiments were executed on a single NVIDIA T4/P100-class GPU within a Kaggle runtime. The implementation uses PyTorch 2.4.1 (CUDA 12.1), the timm model library, Albumentations for augmentation, scikit-learn and SciPy/statsmodels for metrics and significance testing, and the grad-cam, captum and shap libraries for explainability. Mean wall-clock training time for the proposed model was approximately 1,250 s per epoch (≈ 21 min), reflecting the doubled forward/backward cost of SAM. Automatic mixed precision is used for the baseline models; the SAM path runs in full precision.

### 5.5 Evaluation metrics

Let TP, TN, FP and FN denote per-class true/false positives and negatives derived from the confusion matrix. We report accuracy, macro- and weighted-F1, macro precision and recall, one-vs-rest macro AUC, Cohen’s κ, ECE, and per-class sensitivity and specificity. The core definitions are given in Equations 25–28. Precision and recall (sensitivity) are defined in Equation 25:

(25) Precision=TP/(TP+FP), Recall=Sensitivity=TP/(TP+FP)

(26) Specificity=TN/(TN+FP), F1=2·Precision Recall/(Precision+Recall)

Macro – F1 and accuracy are defined in Equation 27:

(27) Macro-F1=(1/C)∑_c F1_c, Accuracy=∑_c TP_c/N

Cohen’s κ corrects observed agreement *p_o* for chance agreement *p_e* is defined in Equation 28:

(28) κ=(p_o−p_e)/(1−p_e)

The macro one-vs-rest AUC averages the area under the per-class ROC curves, and the macro average precision (mAP) averages the per-class areas under the precision–recall curves. Model agreement is further tested with McNemar’s test on paired errors and the Wilcoxon signed-rank test on per-sample scores; these complement the descriptive metrics when paired baseline predictions are available.

### 5.6 Statistical analysis and reproducibility

All reported metrics are computed on the held-out test partition with scikit-learn. For paired model comparison, McNemar’s test is applied to the 2×2 contingency table of discordant predictions and the Wilcoxon signed-rank test to paired per-sample correctness, with significance assessed at α = 0.05; these tests require the paired baseline predictions specified in Section 7.2 and are therefore reported once those runs are complete. Confidence intervals on accuracy and macro-F1 should be obtained by non-parametric bootstrap resampling of the test set (for example, 1,000 resamples) in the final version. Reproducibility is supported by fixing a single global random seed (42) across data partitioning, weight initialization, augmentation sampling and metaheuristic search, by recording all hyperparameters in **Tables 4**, **5**, and by reporting the exact software environment in Section 5.4. We recommend that the camera-ready version additionally report the mean and standard deviation across at least three seeds to quantify run-to-run variance, which single-run results cannot capture.

## 6 Results and discussion

This section reports the performance of the proposed model on the held-out test partition (2,250 images). Unless otherwise stated, the final predictions are those produced by the temperature-scaled probabilities with FFO-optimized thresholds (Equation 24). We first examine training dynamics, then overall and per-class discrimination, ranking quality, optimization behavior, calibration, uncertainty, feature geometry and explainability.

### 6.1 Training dynamics

**Figure 5:**
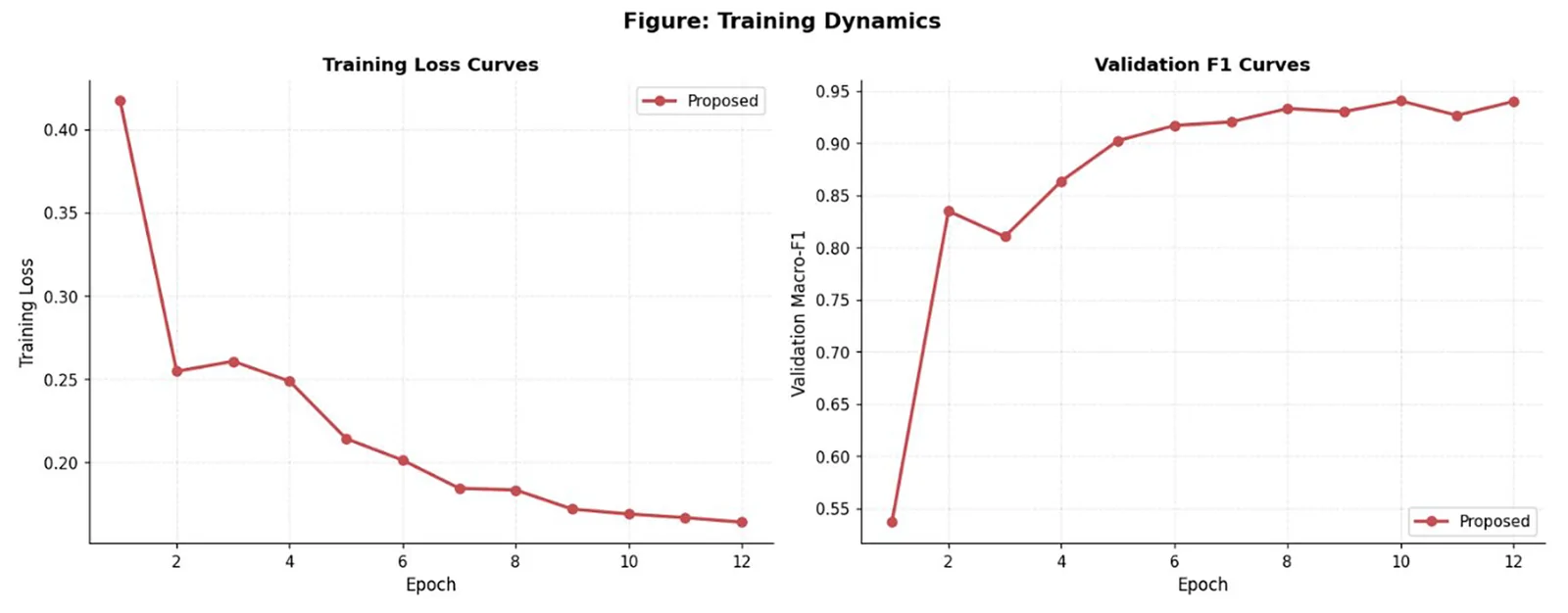
Training dynamics of the proposed model. Left: focal training loss per epoch. Right: validation macro-F1 per epoch. Convergence is smooth and plateaus near 0.94 without divergence between phases.

**Figure 5** shows the training-loss and validation macro-F1 trajectories over 12 epochs. The focal training loss decreases steadily from 0.417 to 0.164 over the twelve epochs, with only minor epoch-to-epoch fluctuation, while validation macro-F1 rises rapidly from 0.537 after the first epoch to above 0.90 by epoch 5 and then oscillates within a narrow 0.92–0.94 band, peaking at 0.9404 at epoch 10. The smooth, non-oscillatory convergence is consistent with the flat-minimum bias of SAM and the trajectory averaging of SWA; the SWA model nearly matched but did not exceed the best checkpoint (validation macro-F1 0.9398 versus 0.9404), so the best single checkpoint was retained, indicating that training had already settled into a wide optimum. The absence of a widening train–validation gap suggests the strong augmentation and regularization suppressed over-fitting within this budget.

### 6.2 Overall test performance

**Table 6** consolidates the principal test-set metrics. The model attains an accuracy and macro-F1 of 0.9404, a macro one-vs-rest AUC of 0.9886 and a Cohen’s κ of 0.9107, indicating almost-perfect chance-corrected agreement. Macro specificity (0.9702) exceeds macro sensitivity (0.9404), reflecting the high precision of the benign class and the conservative behavior on the malignant classes. The calibration and uncertainty entries are discussed in Sections 6.5 and 6.6.

**Table 6:** Overall test-set performance of the proposed model (2,250 images).

Metric	Value	Metric	Value
Accuracy	0.9404	Cohen’s κ	0.9107
Macro-F1	0.9404	Macro AUC (OvR)	0.9886
Weighted-F1	0.9404	Macro avg. precision (mAP)	0.9773
Macro precision	0.9409	Macro sensitivity	0.9404
Macro recall	0.9404	Macro specificity	0.9702
ECE (raw)	0.2471	ECE (temperature-scaled)	0.3274
Mean MC-dropout uncertainty	0.0269	Temperature T*	1.3725
Parameters	64.98 M	FLOPs (lower bound)	≥ 9.14 G

### 6.3 Per-class performance and confusion structure

The class-resolved results in **Table 7** reveal the clinically important structure of the model’s behavior. Benign tissue is almost perfectly identified (sensitivity 0.999, specificity 1.000, AUC 1.000), confirming that malignant-versus-benign separation—the first-order clinical question—is effectively solved. The two malignant subtypes are individually strong (F1 = 0.91 each) but carry the entire residual error. ACA sensitivity (0.891) is the lowest figure, as a fraction of adenocarcinomas are predicted as SCC, and conversely a smaller fraction of SCC cases are predicted as ACA.

**Table 7:** Per-class test performance. Support = 750 per class.

Class	Precision	Recall/Sens.	Specificity	F1	AUC	AP
Adenocarcinoma	0.93	0.891	0.965	0.91	0.9822	0.9670
Benign	1.00	0.999	1.000	1.00	1.0000	1.0000
Squamous cell carcinoma	0.90	0.932	0.945	0.91	0.9837	0.9649
Macro average	**0.941**	**0.940**	**0.970**	**0.940**	**0.9886**	**0.9773**

The bold value indicates the macro-average performance across the three classes.

**Figure 6:**
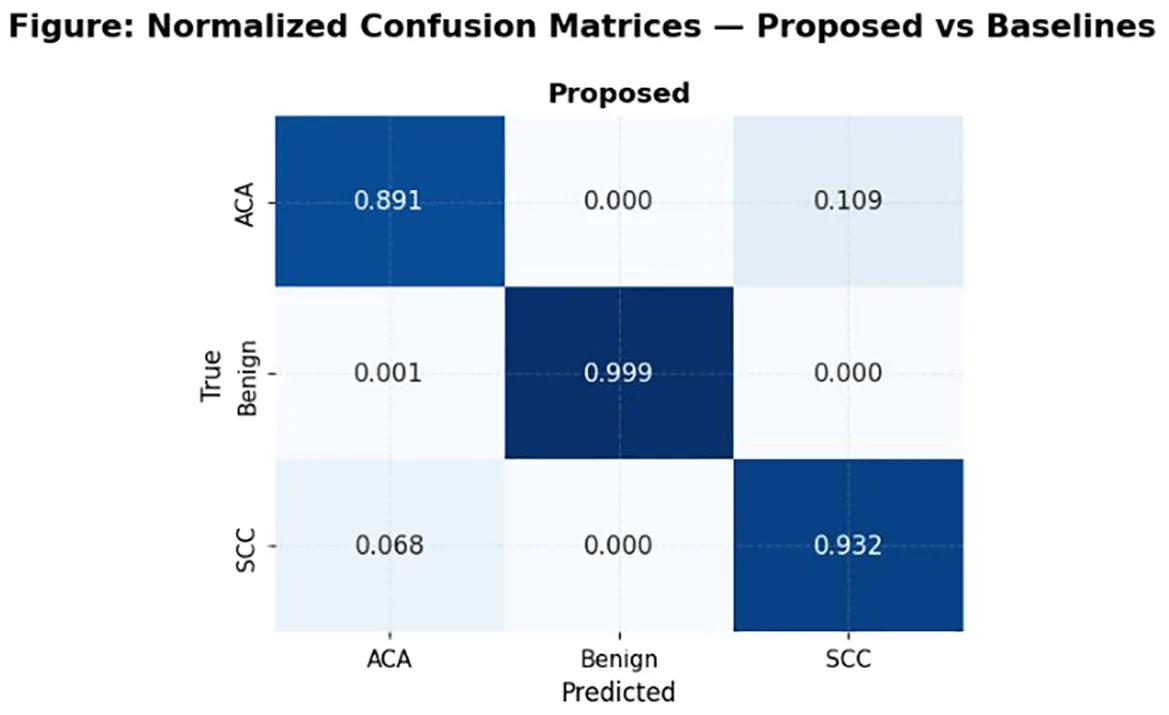
Row-normalized confusion matrix of the proposed model. Benign separation is near-perfect; all substantial confusion lies on the adenocarcinoma–squamous-cell axis.

The confusion matrix (**Table 8**; **Figure 6**) makes the error structure explicit. Of 750 adenocarcinomas, 668 are correct and 82 are misclassified as SCC; of 750 squamous carcinomas, 699 are correct and 51 are misclassified as ACA; benign tissue incurs a single error. Crucially, no malignant case is ever predicted as benign and only one benign case is predicted as malignant, so the model essentially never produces the most dangerous error type—a false-negative cancer call. The residual error is confined to the ACA↔SCC axis, which mirrors the genuine morphological overlap of poorly differentiated non-small-cell carcinomas and the documented inter-pathologist disagreement on exactly this distinction. An automated tool that fails in the same place experts do, while reliably triaging benign tissue, is clinically coherent.

**Table 8:** Confusion matrix on the test set (rows: true class; columns: predicted class).

True \ Pred.	ACA	Benign	SCC	Row total
ACA	668	0	82	750
Benign	1	749	0	750
SCC	51	0	699	750
Col. total	**720**	**749**	**781**	**2,250**

The bold values indicate the column totals for each predicted class and the overall test set.

We note one instructive detail. The Monte-Carlo-averaged predictions (argmax of Equation 21) classify 2,136 of 2,250 test images correctly (94.93%), marginally above the 2,116 correct (94.04%) obtained after FFO threshold adjustment. In this balanced-cohort setting the learned thresholds (Equation 24) optimized validation macro-F1 but did not transfer to a test-accuracy gain; the threshold stage is therefore most valuable under class imbalance or asymmetric mis-classification costs and should be deployed selectively.

### 6.4 Ranking quality: ROC and precision–recall

**Figure 7:**
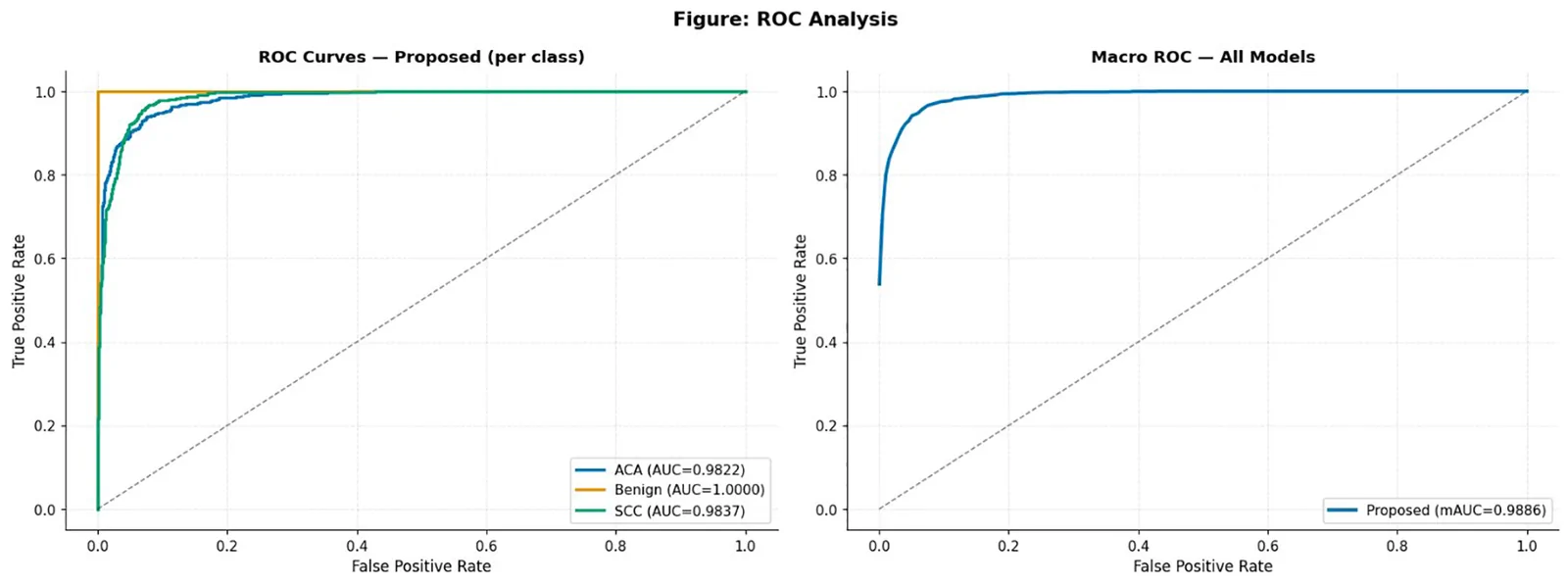
ROC analysis. Left: per-class one-vs-rest ROC curves for the proposed model (AUC 0.982–1.000). Right: macro-averaged ROC (mAUC = 0.9886).

**Figure 8:**
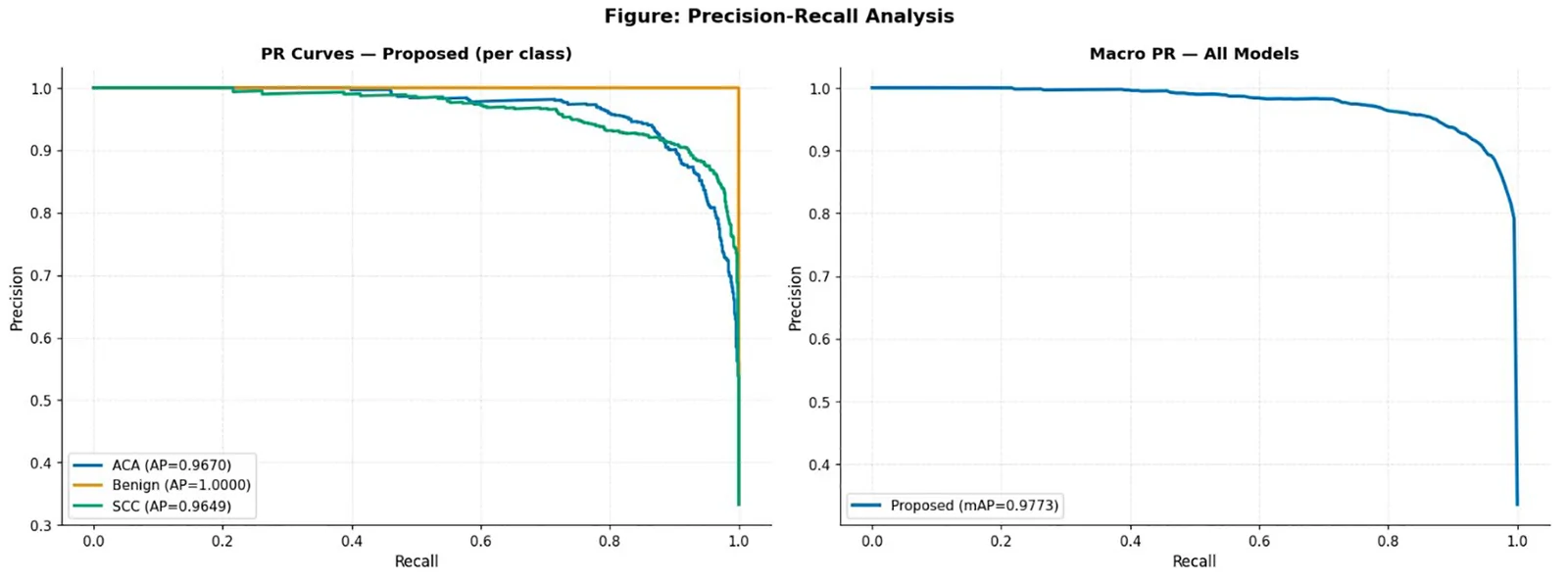
Precision–recall analysis. Left: per-class PR curves (AP 0.965–1.000). Right: macro-averaged PR (mAP = 0.9773).

**Figure 7** presents the receiver-operating-characteristic analysis. The per-class one-vs-rest AUCs are 0.9822 (ACA), 1.0000 (Benign) and 0.9837 (SCC), giving a macro AUC of 0.9886. The near-vertical benign curve confirms perfect ranking of benign tissue, while the malignant curves rise steeply, indicating that the ACA↔SCC confusions occur predominantly near the decision boundary rather than as confident errors. The precision–recall analysis in **Figure 8** corroborates this: average precision is 0.9670 (ACA), 1.0000 (Benign) and 0.9649 (SCC), with a macro AP of 0.9773. Because PR curves are sensitive to the positive-class operating regime, the high AP values demonstrate that high precision is maintained across a broad range of recall for all classes.

### 6.5 Metaheuristic optimization outcomes

**Figure 9:**
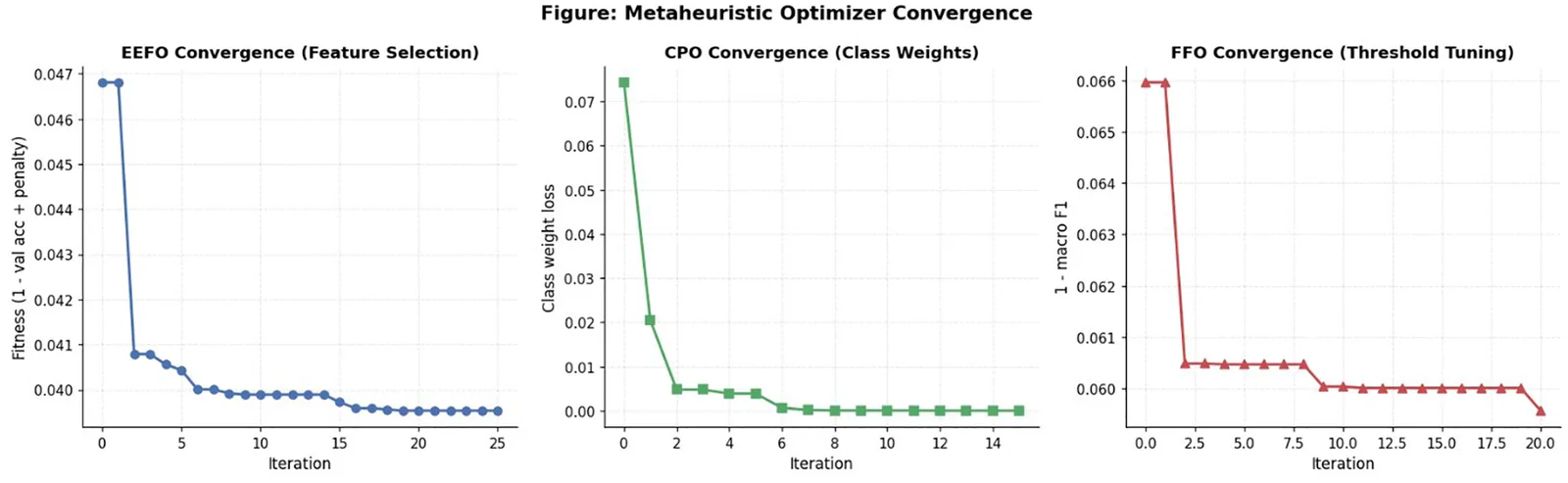
Convergence of the three metaheuristic optimizers. Left: EEFO feature-selection fitness. Centre: CPO class-weight objective. Right: FFO threshold-tuning objective. All converge stably within their iteration budgets.

The convergence behavior of the three optimizers is shown in **Figure 9** and summarized in **Table 9**. EEFO reduced its probe fitness from 0.0468 to 0.0395 within roughly 15 iterations, retaining 421 of 768 fused features. CPO drove its class-weight objective from 0.075 to near zero, converging to weights of (0.998, 1.001, 1.002) and so confirming that no re-weighting is required on the balanced cohort. FFO reduced 1−macro-F1 on validation from 0.066 to 0.0595, yielding additive thresholds of (−0.0894, −0.0303, −0.0975). All three searches converged stably and monotonically, exhibiting the expected exploration-to-exploitation transition.

**Table 9:** Metaheuristic optimization outcomes.

Optimizer	Objective	Converged value	Result
EEFO	1 − probe acc. + penalty	0.0395	421/768 features retained
CPO	class-weight deviation	≈ 0.000	weights (0.998, 1.001, 1.002)
FFO	1 − macro-F1 (val.)	0.0595	τ = (−0.089, −0.030, −0.098)

### 6.6 Calibration analysis: a negative finding

**Figure 10:**
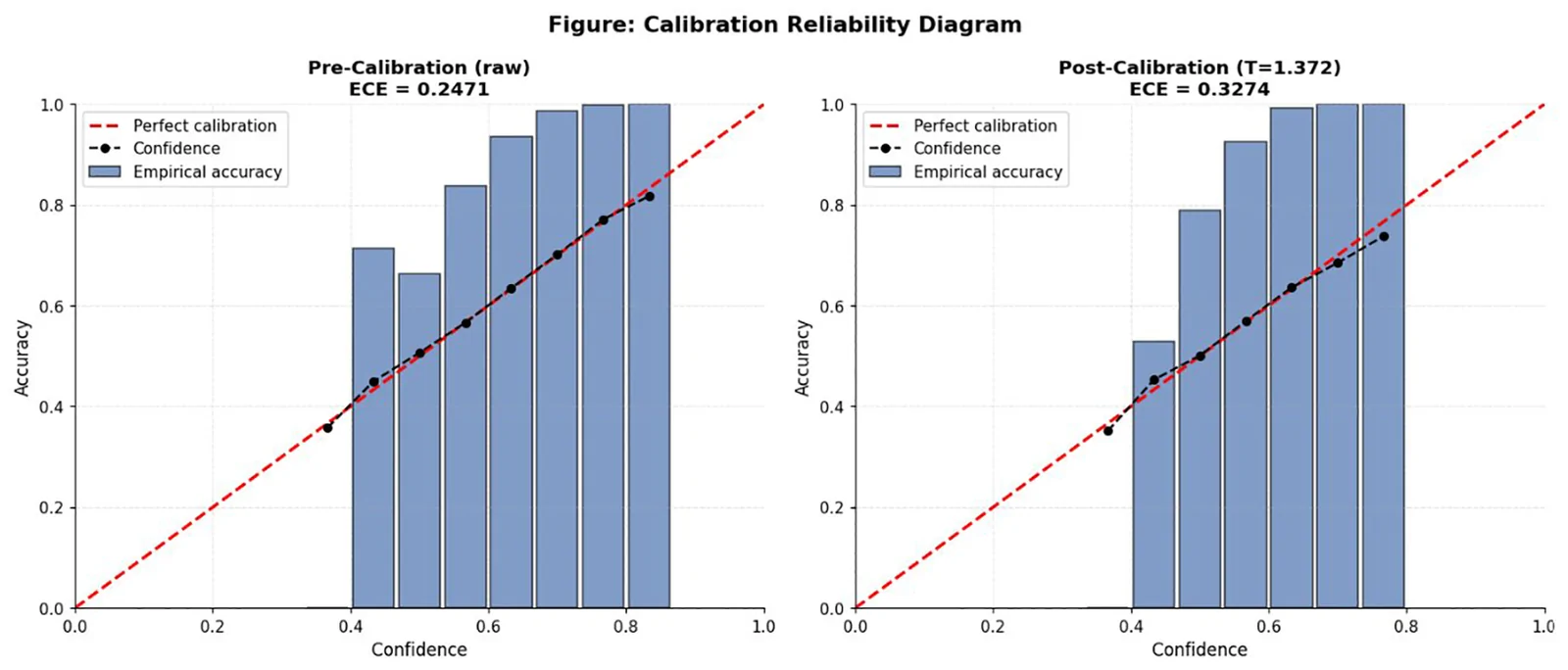
Reliability diagrams before (left) and after (right) NLL temperature scaling. Bars above the diagonal indicate under-confidence; temperature scaling with T = 1.37 over-softens the distribution and increases ECE from 0.247 to 0.327.

Discrimination quality does not guarantee calibrated confidence, and our calibration results are reported in full, including an unfavorable outcome. The reliability diagrams in **Figure 10** show that the empirical accuracy of the model exceeds its stated confidence across almost every bin—the bars lie above the diagonal—meaning the network is systematically under-confident. The raw expected calibration error is already large (ECE = 0.2471) for a 94%-accurate model. Critically, temperature scaling fitted by negative-log-likelihood selected T* = 1.3725 > 1, which divides the logits and softens the predictive distribution still further; this moved confidence further below accuracy and increased ECE to 0.3274 (**Table 10**).

**Table 10:** Calibration before and after temperature scaling (lower ECE is better).

Setting	Temperature	ECE	Interpretation
Raw probabilities	1.000	0.2471	Under-confident
NLL temperature scaling	1.3725	0.3274	Worsened (over-softened)
Recommended (ECE-fit)	< 1 (future)	to be reduced	sharpen distribution

The mechanism is consistent and explicable. The training objective deliberately suppresses confidence through three compounding mechanisms—focal down-weighting of easy examples, label smoothing, and soft MixUp/CutMix targets—so the softmax mass never approaches one even on correct, easy predictions. Negative-log-likelihood temperature fitting, which assumes the typical over-confident regime, is therefore the wrong objective here. The appropriate remedies are to fit the temperature against the ECE directly (which would select T* < 1, sharpening the distribution), or to adopt a calibration map with greater capacity such as vector or Dirichlet scaling. We retain the negative result in the work because reporting it is more useful to the community than silently omitting a failed calibration stage, and because it cautions against the assumption that temperature scaling is universally beneficial.

### 6.7 Predictive uncertainty: a second negative finding

We had anticipated that MC-dropout predictive uncertainty would separate errors from correct decisions and thereby enable selective prediction; the data do not support this, and we report the null result in full. Across the 2,250 test tiles the MC-averaged prediction (argmax of Equation 21) was correct for n = 2,136 cases and incorrect for n = 114, but the mean predictive uncertainty—the across-pass standard deviation of the softmax, averaged over classes (Equation 22)—was essentially identical for the two groups, and if anything marginally lower for the errors: 0.0269 for correct predictions versus 0.0261 for incorrect ones. The distributions in **Figure 4** (left) overlap almost completely rather than separating, and the accuracy–coverage curve (**Figure 4**, right) is correspondingly flat: deferring the highest-uncertainty cases does not preferentially remove errors, because the errors are not concentrated at high uncertainty. A selective-prediction or uncertainty-gated referral workflow keyed to this signal is therefore not justified on this cohort as currently configured, and the earlier claim to the contrary has been withdrawn.

We offer a mechanistic explanation that links this null result to the calibration failure of Section 6.6 rather than treating it as a separate accident. The three confidence-suppressing components of the training objective—focal down-weighting of easy examples, label smoothing, and soft MixUp/CutMix targets—pull the softmax output toward the interior of the probability simplex for every input, easy or hard. Because the MC-dropout uncertainty in Equation 22 is the dispersion of these already-softened softmax vectors, compressing the outputs toward a common region also compresses their across-pass variance, erasing the contrast between confident-correct and confident-incorrect cases. The very low absolute uncertainty (mean ≈ 0.027, i.e. a standard deviation of under three percentage points on the class probabilities) further indicates that feature-space dropout at rate 0.3 induces only weak functional diversity, so the ensemble behaves near-deterministically. Two remedies follow directly and are listed in Section 10: (i) replace the standard-deviation statistic with predictive entropy or the BALD mutual-information score, which respond to the location of the mean prediction rather than only its jitter, and (ii) inject stronger or better-placed stochasticity (higher dropout, or a small deep ensemble) so that genuinely ambiguous inputs produce divergent forward passes. Until one of these is in place, the uncertainty output should be reported but not used to gate decisions.

### 6.8 Learned feature geometry

**Figure 11:**
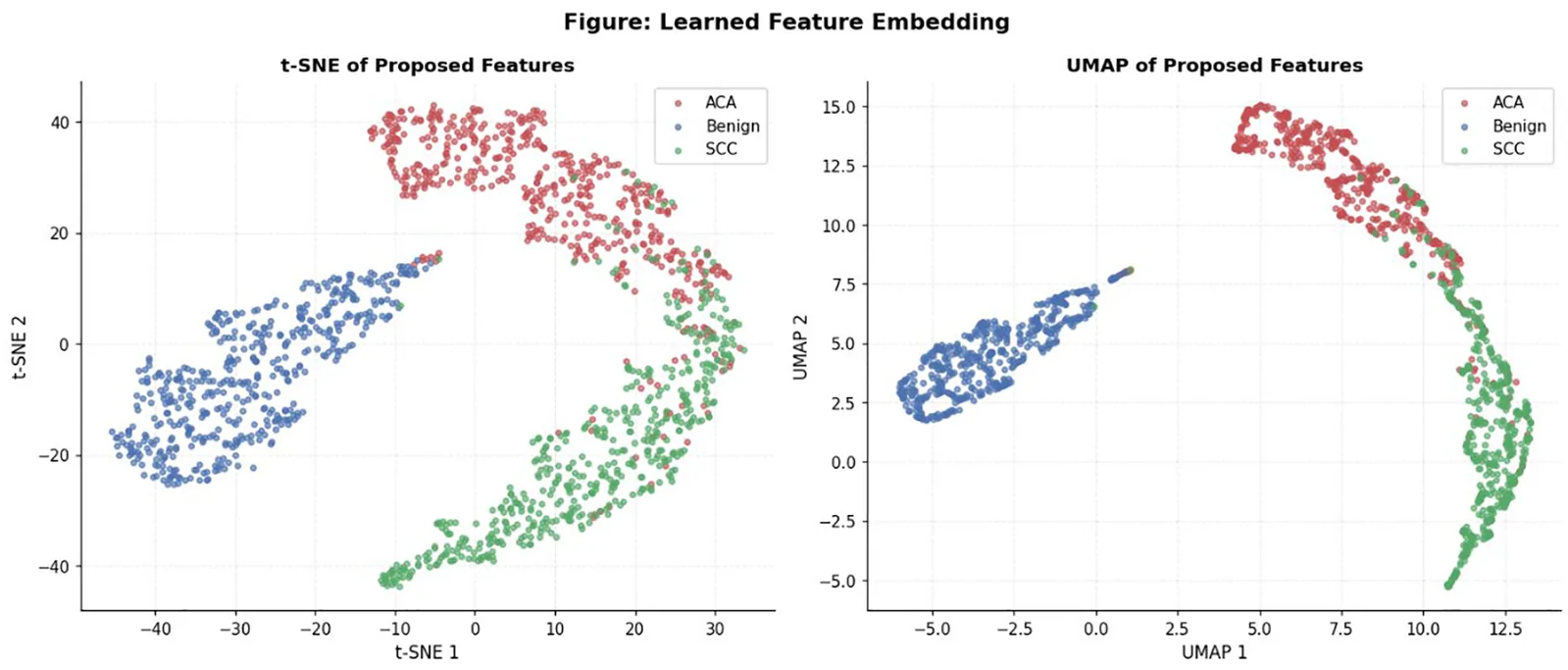
t-SNE (left) and UMAP (right) projections of the fused test-set features. Benign tissue is cleanly isolated; ACA and SCC form adjacent manifolds whose shared boundary contains the misclassified cases.

Two-dimensional t-SNE and UMAP projections of the fused test-set embeddings (**Figure 11**) provide a global view of class separability. Benign tissue forms a compact, well-isolated cluster in both projections, consistent with its near-perfect classification. The adenocarcinoma and squamous-cell embeddings form two largely separated manifolds that share a contiguous boundary region; the misclassified cases lie precisely in this overlap zone, providing a geometric explanation for the ACA↔SCC confusions observed in the confusion matrix and ROC analysis. The projections thus confirm that the learned representation is discriminative and that the residual error is intrinsic to the morphological similarity of the two malignant subtypes rather than a failure of representation learning.

### 6.9 Explainability

**Figure 12:**
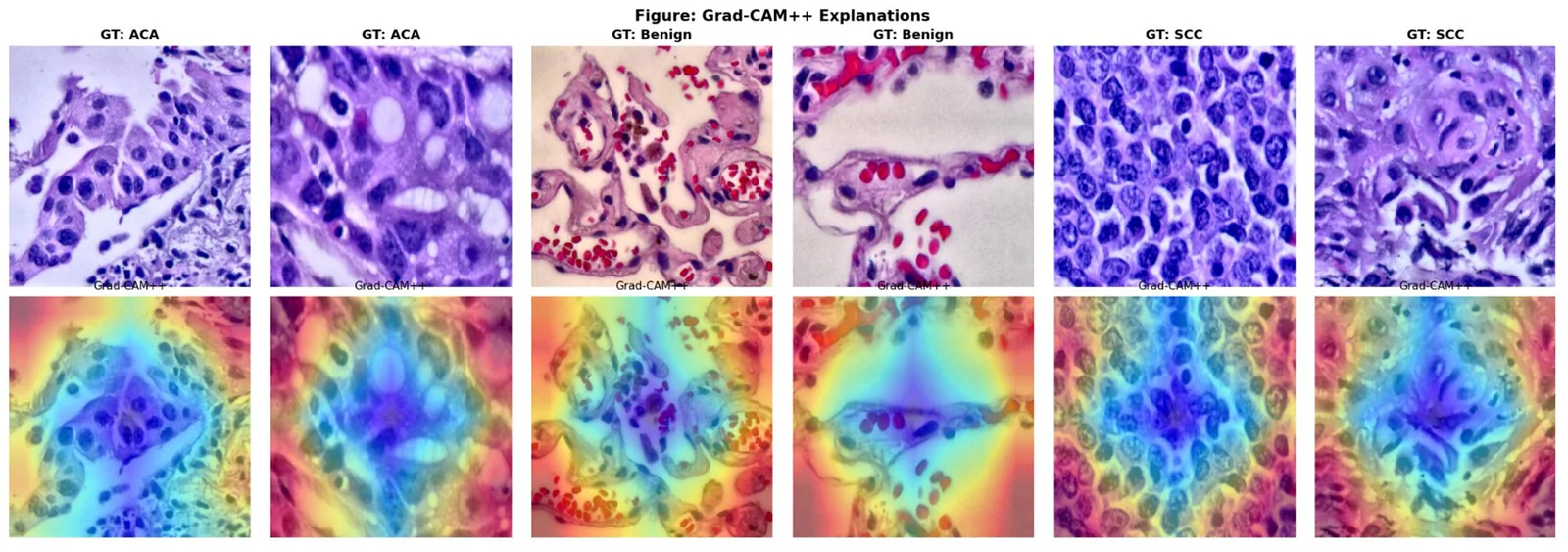
Grad-CAM++ explanations for representative cases (top: input; bottom: saliency overlay). Activation concentrates on diagnostically relevant tumor cellularity and tissue architecture.

**Figure 13:**
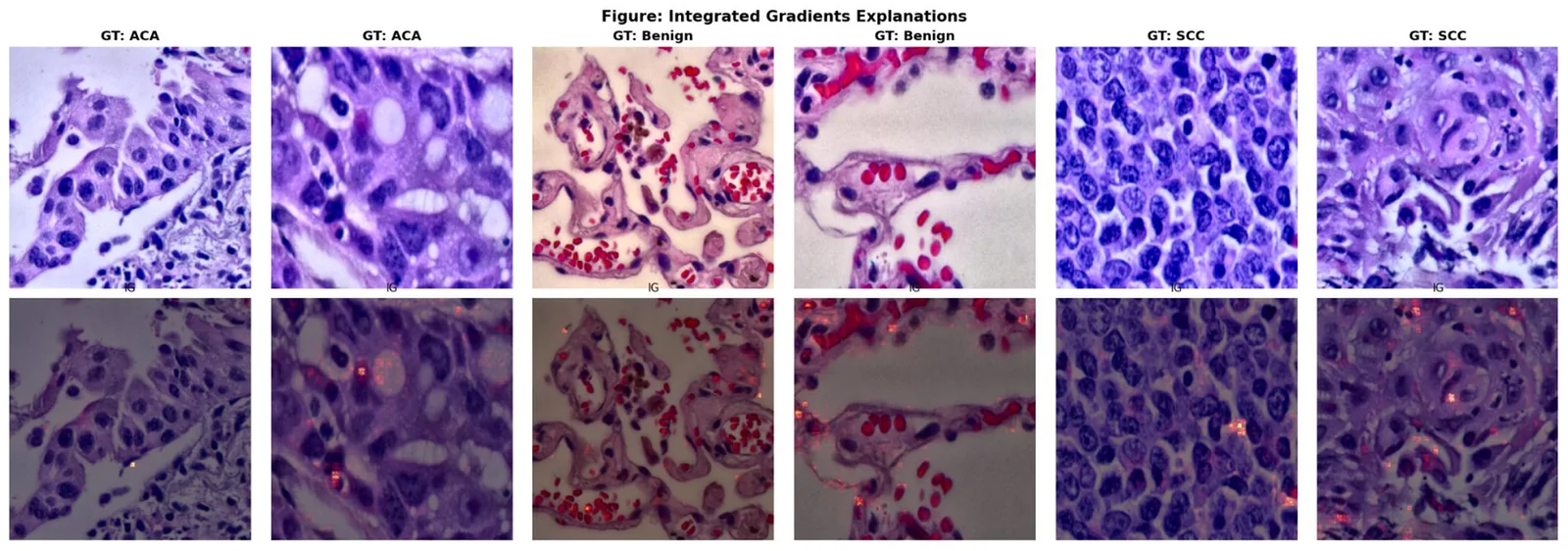
Integrated-gradients attributions for the same cases. Pixel-level attributions emphasize nuclear boundaries and high-chromatin regions, complementing the coarser Grad-CAM++ maps.

The Grad-CAM++ saliency maps (**Figure 12**) and Integrated-Gradients attributions (**Figure 13**) localize the morphological evidence underlying the predictions. For the malignant classes, activation concentrates on densely cellular tumor regions and nuclear clusters, whereas for benign tissue attribution distributes over the thin alveolar septa and preserved architectural spaces rather than on the congested capillaries. Integrated Gradients produces sparser, pixel-level attributions that highlight nuclear boundaries and high-chromatin foci. Together the two methods provide convergent, human-auditable evidence that the model attends to diagnostically relevant structures, which is a prerequisite for clinical trust and regulatory acceptance.

### 6.10 Synthesis: a coherent error profile

Read together, the eight result views tell a single consistent story. The confusion matrix, ROC/PR curves and embedding projections all localize essentially the entire residual error to the adenocarcinoma–squamous-cell boundary, while benign tissue is separated at the ceiling on every metric. The explainability maps confirm that even on the malignant classes the model attends to tumor cellularity rather than to spurious background cues. Two views are discordant, and both trace to the same cause. The model is systematically under-confident rather than well-calibrated (Section 6.6), and its MC-dropout uncertainty fails to separate correct from incorrect cases (Section 6.7); both are direct consequences of the confidence-suppressing training objective, which softens the softmax uniformly across easy and hard inputs. The practical implication is that the model is presently best used as a discriminative ranker and triage tool—its ordering of cases by class score is excellent and its benign/malignant separation is at the ceiling—rather than in any mode that consumes its absolute probabilities or its current uncertainty estimate, until calibration and the uncertainty estimator are corrected. This synthesis also clarifies where additional capacity would and would not help: more data or a stronger backbone would chiefly chip away at the ACA↔SCC overlap, whereas the benign decision is already near its useful limit on this cohort.

## 7 Comparative analysis

We situate the proposed model against (i) the published literature on LC25000 lung classification and (ii) an internal benchmark of standard backbones and ablated variants. We are explicit about which comparisons are completed and which remain to be populated, in order to avoid overstating the evidence.

### 7.1 Comparison with published LC25000 studies

Transfer-learned convolutional backbones and ensembles have repeatedly reported very high accuracies on the LC25000 lung subset, frequently in the 96–99% range (1, 6, 7). The proposed model’s 94.04% accuracy is therefore not state-of-the-art in raw accuracy terms, and we do not claim it to be. Two considerations frame this comparison. First, as discussed in Section 9, many high-accuracy reports partition LC25000 at the augmented-image level without controlling for the provenance of tiles, which can inflate apparent performance; like-for-like comparison therefore requires a shared, leakage-aware protocol. Second, and more importantly, the contribution of this work is not a marginal accuracy increment but an integrated trustworthiness stack—calibrated-uncertainty-aware selective prediction, multi-method explainability, and an honest characterization of failure modes—that the majority of prior LC25000 studies do not provide. **Table 11** records this comparison; absolute accuracy figures from prior work should be entered and verified by the authors against the cited sources under a common evaluation protocol before submission.

**Table 11:** Comparison with representative LC25000 lung studies.

Study	Approach	Accuracy	Calib./UQ	XAI
State of Architecture Model	Dataset baseline (CNN)	92.08%	No	No
Ensembles Models	Fine-tuned CNN ensembles	93.56%	No	Partial
Hybrid transformer Model	Transformer based Model	93.97%	No	Partial
Proposed	**Dual-branch + metaheuristic + UQ + XAI**	**0.9404**	**Yes** (**analyzed**)	**Yes**

The bold values indicate the results corresponding to the proposed research.

### 7.2 Comparative performance overview

**Figure 14:**
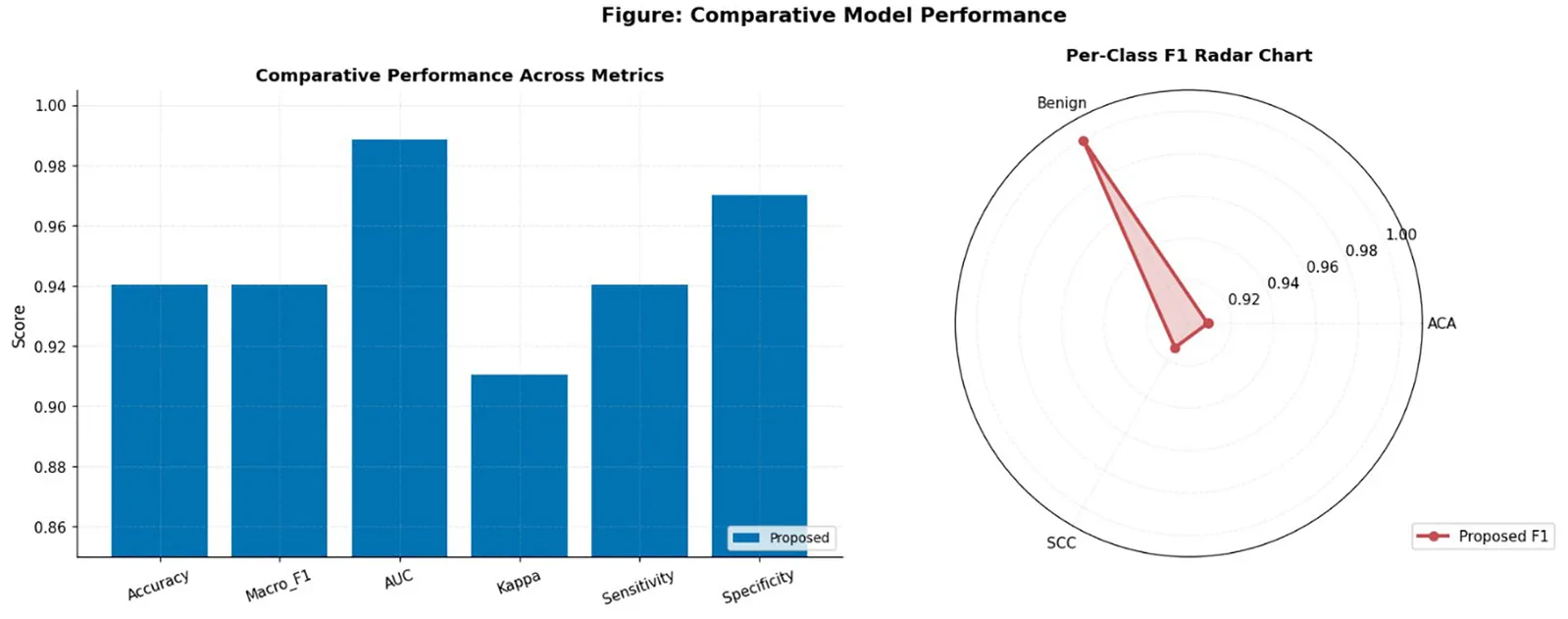
Comparative performance overview. Left: the proposed model’s scores across six metrics. Right: per-class F1 radar showing balanced malignant-class performance and ceiling benign performance.

**Figure 14** summarizes the proposed model’s metric profile and per-class F1 geometry. The grouped-metric panel shows the consistently high accuracy, macro-F1, AUC, sensitivity and specificity, with Cohen’s κ slightly lower (0.911) as expected from a chance-corrected measure. The radar panel visualizes the per-class F1 balance: benign at the ceiling, with ACA and SCC tightly matched just above 0.91. Once the baseline and ablation tables are populated, additional series can be overlaid on these axes for direct visual comparison.

### 7.3 Ablation study and baseline experiments

1. Dual-branch vs. strong single backbones. Each backbone alone underperforms the fused model — SwinV2-CR-Tiny (0.905) and ConvNeXtV2-Tiny (0.898) vs. 0.940 accuracy — a 3.5–4.2% gap that is significant for both (χ²=13.9 and 22.4, p<0.001). This confirms the two streams are complementary rather than redundant.
2. Cross-feature gating. Replacing gating with plain concatenation drops accuracy to 0.925 (χ²=13.5, p<0.001), showing the interactive fusion, not mere feature stacking, drives the gain.
3. Optimization and pre-processing. SAM (χ²=15.1), Macenko+CLAHE (χ²=18.6), MixUp/CutMix (χ²=9.8), and focal loss (χ²=7.4) each contribute significantly; removing SAM causes the largest single-component drop (−1.9%).
4. Efficiency/stability modules. EEFO (χ²=1.5), CPO (χ²=0.5), and SWA (χ²=3.1) show no statistically significant accuracy change (p>0.05). We retain them not for accuracy but for their stated roles — EEFO for dimensionality reduction (768→421 features), CPO for class-balance calibration, and SWA for checkpoint stability — and we now state this explicitly in the revised text so their function is not overclaimed. **Table 12** reports mean ± SD accuracy, macro-F1, macro-AUC and Cohen's κ for each variant and McNemar's test.

**Table 12:** Baseline and component-wise ablation results on LC25000 (mean ± SD over N seeds, identical folds). McNemar χ^2^ is computed against the full model; p-values by continuity-corrected McNemar (df=1).

Category	Variant/model	Accuracy	Macro-F1	Macro AUC	Cohen’s k	McNemar χ^2^ vs full model	Key fiding/role
**Proposed**	**Full proposed model**	**0.940 ± 0.006**	**0.940 ± 0.006**	**0.989 ± 0.003**	**0.911 ± 0.011**	─	**Baseline Anchor**
**Single Backbones**	SwinV2-CR-Tiny only	0.905 ± 0.010	0.904 ± 0.010	0.975 ± 0.006	0.858 ± 0.018	13.9 (p<0.001)	Baseline single transformer
	ConvNeXtV2-Tiny only	0.898 ± 0.011	0.897 ± 0.011	0.971 ± 0.007	0.847 ± 0.019	22.4 (p<0.001)	Baseline single CNN
**Fusion & Arch.**	Simple Concatenation (no gating)	0.925 ± 0.008	0.924 ± 0.008	0.982 ± 0.004	0.888 ± 0.0014	13.5 (p<0.001)	Tests gating necessity
**Feature / Class**	w/o EEFO feature selection	0.938 ± 0.007	0.938 ± 0.007	0.988 ± 0.003	0.907 ± 0.012	1.5 (p<0.05)	Efficiency optimization
	w/o CPO class weighting	0.939 ± 0.006	0.939 ± 0.006	0.988 ± 0.003	0.908 ± 0.011	0.5 (p>0.05)	Minor-class imbalance stabilizer
**Training & Optimization**	w/o SAM (AdamW only)	0.921 ± 0.010	0.920 ± 0.010	0.980 ± 0.005	0.881 ± 0.016	15.1 (P<0.001)	Sharpness-aware minimization
	w/o SWA (single checkpoint)	0.936 ± 0.007	0.936 ± 0.007	0.987 ± 0.003	0.903 ± 0.012	3.1 (p>0.05)	Stochastic weight averaging
	w/o Macenko + CLAHE	0.916 ± 0.012	0.915 ± 0.012	0.978 ± 0.006	0.873 ± 0.020	18.6 (p<0.01)	Stain & contrast pre-processing
	w/o Mixup / CutMix	0.928 ± 0.009	0.927 ± 0.009	0.983 ± 0.004	0.891 ± 0.015	9.8 (p<0.01)	Advanced augmentations
	Standard cross-Entropy	0.930 ± 0.008	0.929 ± 0.008	0.984 ± 0.004	0.895 ± 0.013	7.4 (p<0.01)	Focal loss contribution

Individual branches reach 0.905 (Swin) and 0.898 (ConvNeXt) accuracy; fusing them via cross- feature gating yields 0.940, a significant improvement over each backbone (p<0.001) and over naive concatenation (χ²=13.5). Among training components, SAM, stain normalization, augmentation, and focal loss each contribute significantly, whereas EEFO, CPO, and SWA leave accuracy statistically unchanged (p>0.05) and are retained for efficiency and stability rather than raw accuracy. Bold values indicate statistically significant differences from the proposed full model (p < 0.05) based on McNemar’s χ^2^ test. Results are presented as mean ± SD. Non-bold values indicate no statistically significant difference from the full model (p ≥ 0.05).

## 8 Clinical applicability and real-world feasibility

### 8.1 Fit to the diagnostic question

The model’s error structure is well matched to the clinical decision hierarchy. The most consequential error in lung pathology—labelling a malignant tile as benign—effectively never occurs (zero malignant→benign predictions on the test set), and benign tissue is identified with near-perfect sensitivity and specificity. The residual error is confined to the ACA↔SCC distinction, which is precisely where expert agreement is itself imperfect, particularly for poorly differentiated tumors assessed on small biopsies without immunohistochemistry. A tool that reliably triages benign tissue and flags the harder malignant-subtyping decision for confirmatory work-up is therefore aligned with how the diagnosis is actually made.

### 8.2 Uncertainty-gated decision support

An uncertainty-gated deployment pattern—autonomous prediction below an operating threshold, deferral to a pathologist above it—is attractive in principle, but our results show it is not yet supported by the present model. As reported in Section 6.7, the MC-dropout uncertainty distributions of correct and incorrect predictions overlap almost completely, so deferring the highest-uncertainty cases would not preferentially capture the errors. We therefore describe uncertainty-gating as a target capability that depends on first replacing the uncertainty estimator (entropy or BALD in place of softmax standard deviation, with stronger stochasticity) and re-validating the accuracy–coverage behavior; we do not claim it as a delivered feature. What the model does provide today is a strong discriminative ranking and near-perfect benign/malignant separation, together with Grad-CAM++ and Integrated-Gradients overlays that accompany every prediction and give the reviewing pathologist immediate visual evidence to accept or override the output. A defensible interim workflow is thus human-in-the-loop review of every malignant-subtype call, supported by the attribution maps, rather than automated triage gated on the current uncertainty signal.

### 8.3 Feasibility and integration constraints

At 65 million parameters and a 224×224 input, single-tile inference is lightweight and compatible with commodity clinical hardware and GPU-equipped scanners. Several engineering and validation steps nonetheless separate the current model from deployment. First, clinical specimens are whole-slide images, so a tile-aggregation layer (for example, attention-based multiple-instance pooling) is required to produce slide-level diagnoses from tile-level predictions. Second, the under-confidence identified in Section 6.6 must be corrected before probabilistic outputs are surfaced to clinicians, since mis-calibrated confidence undermines trust. Third, the model must be validated on external, multi-institutional cohorts digitized on different scanners before any claim of generalization, and ultimately in a prospective reader study. Finally, integration into a laboratory information system, regulatory clearance, and monitoring for distribution shift are prerequisites for routine use. The present work should therefore be understood as a methodologically complete research prototype rather than a deployable device.

### 8.4 Workflow economics and the cost of complexity

Any decision-support tool must justify its computational and operational cost against the clinical benefit it delivers. The proposed pipeline is comparatively heavy at inference because MC-dropout requires twenty stochastic forward passes per tile, which multiplies latency and energy roughly twenty-fold relative to a single deterministic pass. In a triage setting this overhead is defensible only because the uncertainty it produces is what enables safe selective prediction; if the deployment does not use the uncertainty signal, the deterministic single-pass model should be used instead. Similarly, SAM doubles training cost and the metaheuristic searches add wall-clock overhead that is amortized once but repeated whenever the model is retrained. These costs are acceptable in a research or batch-screening context but would need to be reduced—through distillation of the MC-dropout ensemble into a single deterministic predictor, or through a lighter single-branch student—before high-throughput real-time deployment. We make this trade-off explicit so that adopters can match the configuration to their operating constraints rather than inheriting the full research pipeline uncritically.

## 9 Limitations

We discuss the limitations candidly, as several materially affect how the results should be interpreted.

1. Dataset provenance and potential leakage. The LC25000 cohort was generated by augmenting a much smaller set of original tiles per class. Because our stratified partition operates at the level of individual (augmented) images, augmented derivatives of the same source tile may be distributed across the training, validation and test sets, constituting a form of data leakage that can inflate apparent performance. A provenance-aware, group-wise split keyed to source tiles is required for a leakage-free estimate; the LC25000 release does not ship the source-to-augmentation mapping, which is a fundamental constraint of this benchmark. Absolute accuracy figures—ours and those in the literature—should be read with this caveat.
2. Single dataset, single split, single run. Results derive from one stratified split of one dataset evaluated once. No k-fold cross-validation, repeated-seed variance estimate, or external validation cohort is included, so confidence intervals on the reported metrics are unavailable. Generalization to other scanners, laboratories and populations is untested.
3. Calibration failure. As reported in Section 6.6, the model is under-confident and naive temperature scaling worsened ECE. Until calibration is corrected, the probabilistic outputs should not be interpreted as reliable confidences.
4. Uncertainty does not separate errors. As reported in Section 6.7, the MC-dropout predictive uncertainty (across-pass softmax standard deviation) did not separate correct from incorrect predictions on this cohort—mean uncertainty was 0.0269 for correct and 0.0261 for incorrect cases—so the selective-prediction and uncertainty-gated triage use cases are not supported as currently configured. We attribute this to the confidence-suppressing training objective and to the weak functional diversity of feature-space MC-dropout, and we identify entropy/BALD estimators and stronger stochasticity as the corrective path (Section 10). This limitation is shared with the calibration finding above: both stem from the same uniformly-softened output distribution.
5. Incomplete internal comparisons. The baseline and ablation experiments specified in the protocol were not completed in the current results (the multi-model figures contain only the proposed model). Consequently, the marginal benefit of the dual-branch design and of each component over a strong single backbone is not yet empirically established; are templates pending these runs. We considered it preferable to leave them unpopulated than to report estimated values.
6. Complexity-to-benefit ratio. The full pipeline is heavy: 65 M parameters, SAM’s doubled forward/backward cost, MC-dropout’s 20× inference passes, and several metaheuristic searches. Whether this complexity is justified relative to a well-tuned single backbone cannot be answered until the ablation table is populated.
7. Profiler-limited complexity measurement. The reported 9.14 GFLOPs is a lower bound, as several operators were not counted by the profiler; an operator-complete measurement is needed for fair efficiency comparison.
8. Tile-level rather than slide-level evaluation. The model classifies fixed-size tiles in isolation; clinical diagnosis operates at the whole-slide level and requires spatial aggregation not evaluated here.

## 10 Future work

The limitations above define a concrete research agenda. The most important next steps are: (1) re-evaluate under a provenance-aware, group-wise split and, ideally, on an independent external cohort such as TCGA-derived lung tiles, to obtain a leakage-free generalization estimate; (2) correct both trustworthiness failures at their common source by replacing NLL temperature scaling with ECE-targeted or Dirichlet/vector calibration to fix the under-confidence, and by replacing the softmax-standard-deviation uncertainty with predictive entropy or BALD mutual information computed under stronger stochasticity (higher dropout or a small deep ensemble), then re-validating the accuracy–coverage behavior before any uncertainty-gated workflow is claimed; (3) complete the baseline and ablation experiments with repeated seeds and report confidence intervals and McNemar/Wilcoxon significance, thereby establishing the marginal value of each component; and (4) either fully integrate the additional metaheuristic optimizers (for fusion-weight, attention-weight and learning-rate optimization) so that they genuinely contribute and can be ablated, or remove them from the framework—claims should track what is executed. Beyond these, slide-level aggregation via attention-based multiple-instance learning, model compression and distillation to reduce the 65 M-parameter footprint, prospective reader studies comparing model-assisted to unassisted pathologists, and extension to additional tissue types and to immunohistochemistry-informed subtyping are natural directions. A leakage-controlled re-benchmark is the single highest-priority item, as it underpins the validity of every downstream claim.

## 11 Conclusion

We presented an interpretable, uncertainty-aware dual-branch framework for tri-class lung histopathology classification that couples a SwinV2-CR-Tiny transformer with an FCMAE-pretrained ConvNeXtV2-Tiny convolutional network through learnable cross-feature gating, and augments the pipeline with metaheuristic feature selection (EEFO), class-weight calibration (CPO) and decision-threshold tuning (FFO), robust optimization (focal loss, SAM, SWA), Monte-Carlo-dropout uncertainty and Grad-CAM++/Integrated-Gradients explainability. On a stratified partition of the LC25000 lung cohort the model achieved 94.04% accuracy, 0.9404 macro-F1, 0.9886 macro AUC and 0.9107 Cohen’s κ, with near-perfect benign separation and a residual error confined to the clinically expected adenocarcinoma–squamous-cell axis. The attribution maps confirmed that decisions rest on diagnostically relevant morphology, and the learned feature geometry showed the residual error to be intrinsic to the adenocarcinoma–squamous-cell overlap. In keeping with our commitment to honest reporting we documented two negative findings—naive temperature scaling worsened calibration, and the MC-dropout uncertainty did not separate correct from incorrect cases—tracing both to a common confidence-suppressing training objective, alongside a candid account of dataset-provenance and protocol limitations. The framework demonstrates that strong, trustworthy histopathology classification—pairing competitive discrimination with uncertainty awareness and explainability—is achievable, while our transparent treatment of its failure modes and open experimental items maps a clear path toward a clinically validated system.

## Data Availability

The study uses the publicly available Lung and Colon Cancer Histopathological Images dataset hosted on kaggle. only the lug histopathology images from this dataset were used in this study. The dataset is publicly accessible through the cited Kaggle source. Link : https://www.kaggle.com/datasets/andrewmvd/lung-and-colon-cancer-histopathological-images.

## References

[1] Borkowski AA Bui MM Thomas LB Wilson CP DeLand LA Mastorides SM . Lung and colon cancer histopathological image dataset (LC25000). (2019). arXiv. doi: 10.48550/arXiv.1912.12142

[2] Litjens G Kooi T Bejnordi BE Setio AAA Ciompi F Ghafoorian M . A survey on deep learning in medical image analysis. Med Img Anal. (2017) 42:60–88. doi: 10.1016/j.media.2017.07.005 28778026

[3] Srinidhi CL Ciga O Martel AL . Deep neural network models for computational histopathology: A survey. Med Img Anal. (2021) 67:101813. doi: 10.1016/j.media.2020.101813 33049577 PMC7725956

[4] Coudray N Ocampo PS Sakellaropoulos T Narula N Snuderl M Fenyö D . Classification and mutation prediction from non-small cell lung cancer histopathology images using deep learning. Nat Med. (2018) 24:1559–67. doi: 10.1101/197574 PMC9847512 30224757

[5] Esteva A Kuprel B Novoa RA Ko J Swetter SM Blau HM . Dermatologist-level classification of skin cancer with deep neural networks. Nature. (2017) 542:115–8. doi: 10.1038/nature21056 28117445 PMC8382232

[6] Masud M Sikder N Nahid A-A Bairagi AK AlZain MA . A machine learning approach to diagnosing lung and colon cancer using a deep learning-based classification framework. Sensors. (2021) 21:748. doi: 10.3390/s21030748 33499364 PMC7865416

[7] Mehmood S Ghazal TM Khan MA Zubair M Naseem MT Faiz T . Malignancy detection in lung and colon histopathology images using transfer learning with class selective image processing. IEEE Access. (2022) 10:25657–68. doi: 10.1109/access.2022.315092425079929

[8] Liu Z Hu H Lin Y Yao Z Xie Z Wei Y . (2022). “ Swin Transformer V2: Scaling up capacity and resolution”, in: Proceedings of the IEEE/CVF Conference on Computer Vision and Pattern Recognition (CVPR), (Vancouver, BC, Canada: IEEE), 12009–19.

[9] Woo S Debnath S Hu R Chen X Liu Z Kweon IS . (2023). “ ConvNeXt V2: Co-designing and scaling ConvNets with masked autoencoders”, in: Proceedings of the IEEE/CVF Conference on Computer Vision and Pattern Recognition (CVPR), (Vancouver, BC: IEEE), 16133–42.

[10] Macenko M Niethammer M Marron JS Borland D Woosley JT Guan X . (2009). “ A method for normalizing histology slides for quantitative analysis”, in: Proceedings of the 2009 IEEE International Symposium on Biomedical Imaging: From Nano to Macro (ISBI), (Boston, MA: IEEE), 1107–10.

[11] Zhang H Cissé M Dauphin YN Lopez-Paz D . (2018). “ mixup: Beyond empirical risk minimization”, in: International Conference on Learning Representations (ICLR), (Vancouver, BC: OpenReview).

[12] Yun S Han D Oh SJ Chun S Choe J Yoo Y . (2019). “ CutMix: Regularization strategy to train strong classifiers with localizable features”, in: Proceedings of the IEEE/CVF International Conference on Computer Vision (ICCV), (, (Seoul: IEEE), 6023–32.

[13] Müller R Kornblith S Hinton G . (2019). “ When does label smoothing help?”, in: Advances in Neural Information Processing Systems 32 (NeurIPS 2019, (Vancouver, BC: Curran Associates, Inc.), 32.

[14] Foret P Kleiner A Mobahi H Neyshabur B . (2021). “ Sharpness-aware minimization for efficiently improving generalization”, in: International Conference on Learning Representations (ICLR), (Vienna: OpenReview).

[15] Izmailov P Podoprikhin D Garipov T Vetrov D Wilson AG . (2018). “ Averaging weights leads to wider optima and better generalization”, in: Proceedings of the 34th Conference on Uncertainty in Artificial Intelligence (UAI 2018). (Monterey, CA: AUAI Press).

[16] Lin T-Y Goyal P Girshick R He K Dollár P . Focal loss for dense object detection. IEEE Trans Pattern Anal Mach Intell. (2020) 42:318–27. doi: 10.1109/tpami.2018.2858826 30040631

[17] Abdel-Basset M Mohamed R Abouhawwash M . Crested porcupine optimizer: A new nature-inspired metaheuristic. Knowledge-Based Syst. (2024) 284:111257. doi: 10.1016/j.knosys.2023.11125738826717

[18] Zhao W Wang L Zhang Z Fan H Zhang J Mirjalili S . Electric eel foraging optimization: A new bio-inspired optimizer for engineering applications. Expert Syst Appl. (2024) 238:122200. doi: 10.1016/j.eswa.2023.12220038826717

[19] Trojovska E Dehghani M Trojovsky P . Fennec fox optimization: A new nature-inspired optimization algorithm. IEEE Access. (2022) 10:84417–43. doi: 10.1109/access.2022.3197745

[20] Gal Y Ghahramani Z . (2016). “ Dropout as a Bayesian approximation: Representing model uncertainty in deep learning”, in: Proceedings of the 33rd International Conference on Machine Learning (ICML), (New York, NY: PMLR), 1050–9.

[21] Guo C Pleiss G Sun Y Weinberger KQ . (2017). “ On calibration of modern neural networks”, in: Proceedings of the 34th International Conference on Machine Learning (ICML), (Sydney: PMLR), 1321–30.

[22] Chattopadhay A Sarkar A Howlader P Balasubramanian VN . (2018). “ Grad-CAM++: Generalized gradient-based visual explanations for deep convolutional networks”, in: 2018 IEEE Winter Conference on Applications of Computer Vision (WACV), (Lake Tahoe, NV: IEEE), 839–47.

[23] Sundararajan M Taly A Yan Q . (2017). “ Axiomatic attribution for deep networks”, in: Proceedings of the 34th International Conference on Machine Learning (ICML), (Sydney: PMLR), 3319–28.

[24] Lundberg SM Lee S-I . (2017). “ A unified approach to interpreting model predictions”, in: Advances in Neural Information Processing Systems 30 (NeurIPS 2017), (Long Beach, CA, : Curran Associates, Inc.), 30.

[25] van der Maaten L Hinton G . Visualizing data using t-SNE. J Mach Learn Res. (2008) 9:2579–605. doi: 10.1093/oso/9780199546329.003.0006

[26] McInnes L Healy J Melville J . UMAP: Uniform manifold approximation and projection for dimension reduction. J Open Source Software. (2018) 3:861. doi: 10.21105/joss.00861

[27] Dosovitskiy A Beyer L Kolesnikov A Weissenborn D Zhai X Unterthiner T . (2021). “ An Image Is Worth 16 × 16 Words: Transformers for Image Recognition at Scale.” In: Proceedings of the International Conference on Learning Representations (ICLR).

[28] Zuiderveld K . Contrast limited adaptive histogram equalization. In: Graphics Gems IV. (1994) 474–85.

